# Optimising summary infographics for clinical practice guidelines on child health: qualitative user testing study with healthcare workers in Malawi, Nigeria and South Africa

**DOI:** 10.1136/bmjopen-2026-122446

**Published:** 2026-10-05

**Authors:** Tandekile Lubelwana Hafver, Frankie Achille, Valerie Reinthaler, Sumanth Nagraj, Susan Banda, Willem Odendaal, Idriss I Kallon, Moriam Chibuzor, Dachi Arikpo, Roselyn Chipojola, Emmanuel Effa, Nyanyiwe Masingi Mbeye, Tamara Kredo, Trudy Desirie D Leong, Simon Lewin, Amanda Salomé Brand, Claire Glenton, Sara Cooper, Bey-Marrié Schmidt, Susan Kyomuhendo Munabi-Babigumira, Per O Vandvik, Thomas Agoritsas, Nicolas Delvaux

**Affiliations:** 1 MAGIC Evidence Ecosystem Foundation, Oslo, Norway; 2 School of Public Health and Family Medicine, Kamuzu University of Health Sciences, Blantyre, Southern Region, Malawi; 3 HIV and Other Infectious Diseases Research Unit, Medical Research Council of South Africa, Cape Town, South Africa; 4 Department of Psychiatry, Stellenbosch University, Stellenbosch, WC, South Africa; 5 Health Systems Research Unit, South African Medical Research Council, Cape Town, Western Cape, South Africa; 6 Division of Epidemiology and Biostatistics, Stellenbosch University, Stellenbosch, South Africa; 7 Cochrane Nigeria, Institute of Tropical Diseases Research and Prevention, University of Calabar Teaching Hospital, Calabar, Nigeria; 8 Evidence Informed Decision-Making Centre, Kamuzu University of Health Sciences, Blantyre, Malawi; 9 Cochrane South Africa, South African Medical Research Council, Cape Town, Western Cape, South Africa; 10 Division of Clinical Pharmacology, Department of Medicine, Stellenbosch University, Stellenbosch, Western Cape, South Africa; 11 Norwegian University of Science and Technology, Trondheim, Norway; 12 Centre for Evidence-based Health Care, Department of Global Health, Faculty of Medicine and Health Sciences, Stellenbosch University, Stellenbosch, Western Cape, South Africa; 13 Department of Health and Functioning, Western Norway University of Applied Sciences, Bergen, Norway; 14 Department of Behavioural Medicine, Institute of Basic Medical Sciences, Faculty of Medicine, University of Oslo, Oslo, Oslo, Norway; 15 Division of General Internal Medicine & Division of Clinical Epidemiology, Geneva University Hospitals, Geneva, Geneva, Switzerland; 16 Department of Health Research Methods, Evidence, and Impact, McMaster University, Hamilton, Ontario, Canada; 17 Department of Public Health and Primary Care, KU Leuven, Leuven, Flanders, Belgium

**Keywords:** PAEDIATRICS, Implementation Science, Africa South of the Sahara

## Abstract

**Abstract:**

**Objectives:**

To optimise the dissemination of clinical practice guideline (CPG) summary infographics on newborn and child health to healthcare workers (HCWs) in Malawi, Nigeria and South Africa.

**Design:**

Qualitative user testing using think-aloud interviews with local HCWs to identify their preferences for and barriers to using CPG summary infographics.

**Participants:**

26 HCWs involved in newborn and child healthcare, including participants from Lilongwe (n=8), Calabar (n=10) and Cape Town (n=8).

**Interventions:**

Draft CPG summary infographics on newborn and child health topics explored by participants during interviews.

**Primary and secondary outcomes:**

Participants’ perceptions of the infographics in terms of findability, usability, understandability, desirability, identification, accessibility, credibility, usefulness and value.

**Results:**

Most participants found the infographics easy to understand and appreciated the visual design. However, participants had difficulty interpreting the strength of recommendations and were sometimes unclear about the target population and distinction between interventions and comparators. Navigation and the use of medical terminology also presented challenges, while some images did not reflect the local clinical environments. Perceptions of usefulness varied across guideline topics and countries.

**Conclusions:**

User testing identified important barriers to the use of CPG summary infographics. Feedback informed revisions to the infographics before final dissemination, including clarifying the meaning of recommendation strength, adjusting terminology, bringing key content to the foreground and refining imagery. Early engagement with end users in the development of CPG dissemination products can help ensure that recommendations are clearly conveyed and that the content and visuals are appropriate to local clinical contexts and needs.

**STRENGTHS AND LIMITATIONS OF THIS STUDY**

- Used qualitative user testing interviews with a ‘think-aloud’ approach to explore user experiences in real time.
- Included healthcare workers from three sub-Saharan African countries, capturing diverse implementation contexts.
- Tested draft infographics based on locally adapted clinical practice guidelines.
- Included a range of healthcare workers involved in newborn and child care.
- Small sample from selected hospitals may limit transferability to other settings.

## Background

 Clinical practice guidelines (CPGs) are critical tools that support healthcare workers (HCWs) in making decisions about appropriate care for specific clinical circumstances,^1^ bridging the gap between best available evidence and actual practice.^2^ This gap, often termed ‘the quality chasm’^3^ is likely even greater in resource-constrained settings.^4^ In response, there has been growing emphasis on developing a better-integrated evidence ecosystem, or learning health system, to incorporate, contextualise and disseminate new evidence for implementation.^5 6^

If research is not accessible and usable, it cannot benefit clinicians and patients.^7 8^ For clinical information to impact care and outcomes, it must reach target users, resonate cognitively and emotionally with them and be applied in practice.^9 10^ Accordingly, the effectiveness of CPGs hinges on how they are disseminated and how easily they can be accessed, understood and applied.

Infographics—visual summaries of CPGs designed to communicate key findings to end users—may increase clinical impact by conveying complex medical information efficiently^11^ and facilitating sharing on social media and digital platforms.^12^ User testing of CPG summary infographics with general practitioners in Belgium and Switzerland found that infographics helped users quickly grasp key elements of recommendations and were seen as valuable tools for CPG translation and implementation.^13 14^ However, evidence is limited on how HCWs in resource-constrained settings experience and use such CPG summary infographics.

As part of the Global Evidence, Local Adaptation (GELA) project^15^ whose overarching goal was to strengthen decision makers’ capacity to use global research evidence to develop locally contextualised CPGs, this study aimed to optimise CPG summary infographics on newborn and child health for use by HCWs in Malawi, Nigeria and South Africa. We conducted user testing interviews with local HCWs to identify content and design features that facilitate or hinder use, informing refinement of the infographics before final dissemination.

## Methods

### Study design

The GELA project produced CPGs on selected newborn and child health topics in Malawi, Nigeria and South Africa. Figure 1 illustrates how global evidence was adapted to local context-specific recommendations. As part of optimising dissemination (blue box in the figure), web-based summary infographics were created for each of the CPGs, with tailored versions to meet the different information needs of policy/decision makers, HCWs and patients/parents/caregivers. User testing interviews were then conducted with HCWs to assess their user experiences and collect feedback to adjust the contents and designs of the final versions. User testing is a well-established method within web design research.^16^

**Figure 1:**
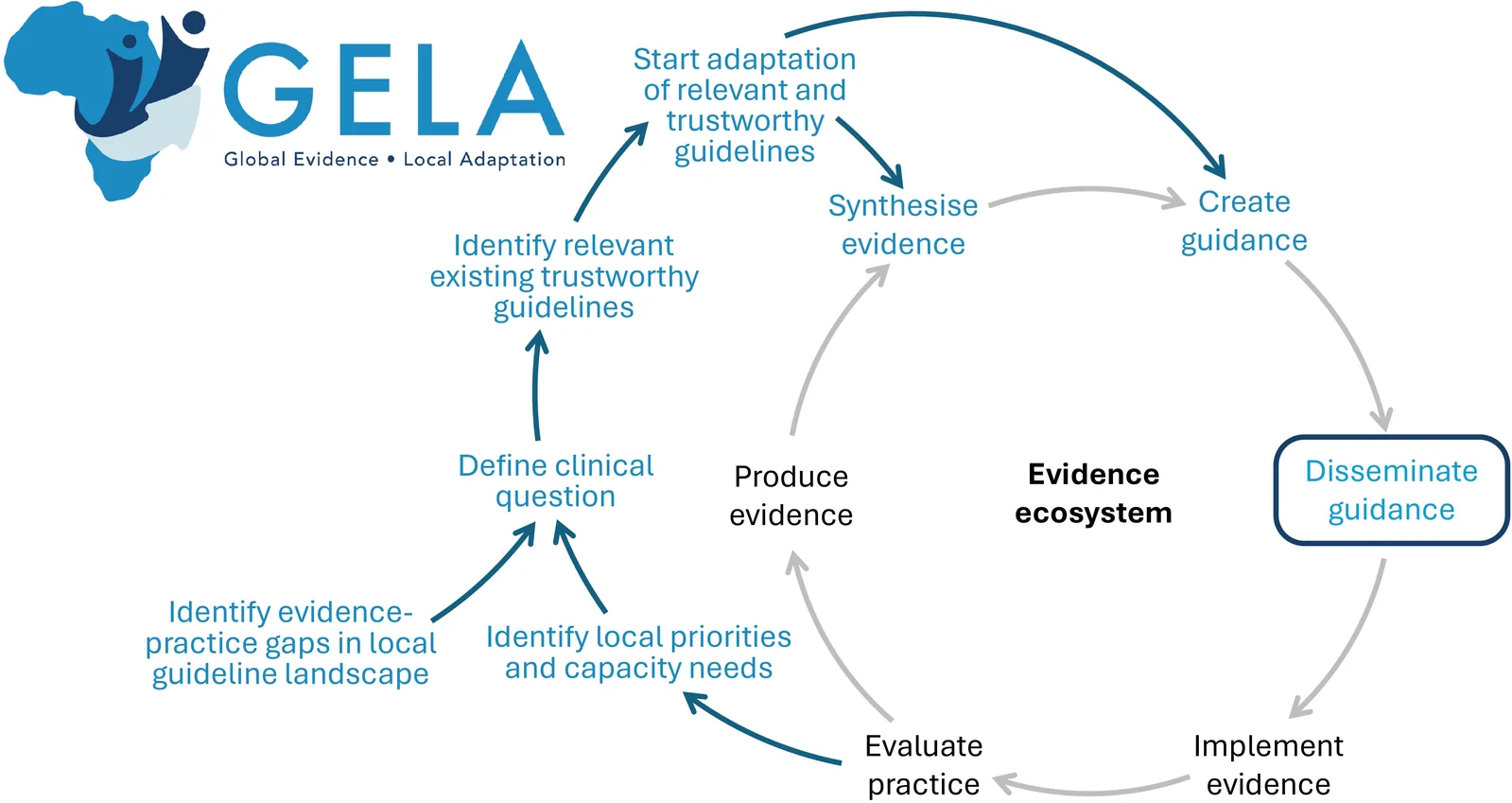
Illustration of how the GELA project and user testing of infographics fits into the evidence ecosystem (figure adapted from Vandvik and Brandt)^5^. Blue activities were covered in GELA, focus highlighted with a box.

This is a descriptive qualitative study using think-aloud user testing interviews based on a user test toolkit^17^ that has previously been employed for user testing a number of evidence products, including other CPG summary infographics.^13 14 18–22^

We followed the Standards for Reporting Qualitative Research (http://www.equator-network.org/reporting-guidelines/srqr/, see online supplemental file 1).

### Intervention tested

The GELA project^15^ adapted and contextualised evidence-informed recommendations on selected topics within newborn and child health in Malawi, Nigeria and South Africa (table 1). These guidelines are available from Cochrane Africa (https://africa.cochrane.org/projects/GELA) and in the MAGICapp (https://app.magicapp.org/#/guidelines/org=gela). For each of the topics, accompanying summary infographics were developed (electronic versions available here https://portal.magicevidence.org/gela/, printed versions in online supplemental files 4–7). Note that only the versions for HCWs were user tested.

**Table 1:** The topics of the CPG summary infographics tested in the three countries

Country	Malawi	South Africa	Nigeria
Topics	Early versus delayed enteral nutrition in critically ill children under 12 years of age	Family-centred support and post-discharge preparation interventions for families with preterm and low birth weight infants	Healthcare worker-related multicomponent interventions for infection prevention and control
			Early versus delayed enteral feeding in preterm and low birth weight infants

CPG, clinical practice guideline.

The infographic design process included brainstorming, stakeholder feedback and co-creation, with iterations based on user testing feedback. The infographics were developed using standard web technologies (HTML, CSS and JavaScript) with responsive and interactive features (eg, collapsible sections) to ensure usability across devices. This is particularly important in low-resource African settings, where computer access is limited but smartphones are widely used. Static versions of the infographics were also made to facilitate sharing in emails, messages, social media and print. All illustrations were generated using ChatGPT as part of the overall design process, with careful review.

We conducted three sets of interviews, one set in each country (figure 2). While the topics differed between the countries (see table 1), the overall designs of the infographics remained similar, allowing much of the learnings to be transferred across infographics and settings. We made minor refinements to infographics between each set of interviews, and finalised adjustments after completing all three rounds.

**Figure 2:**
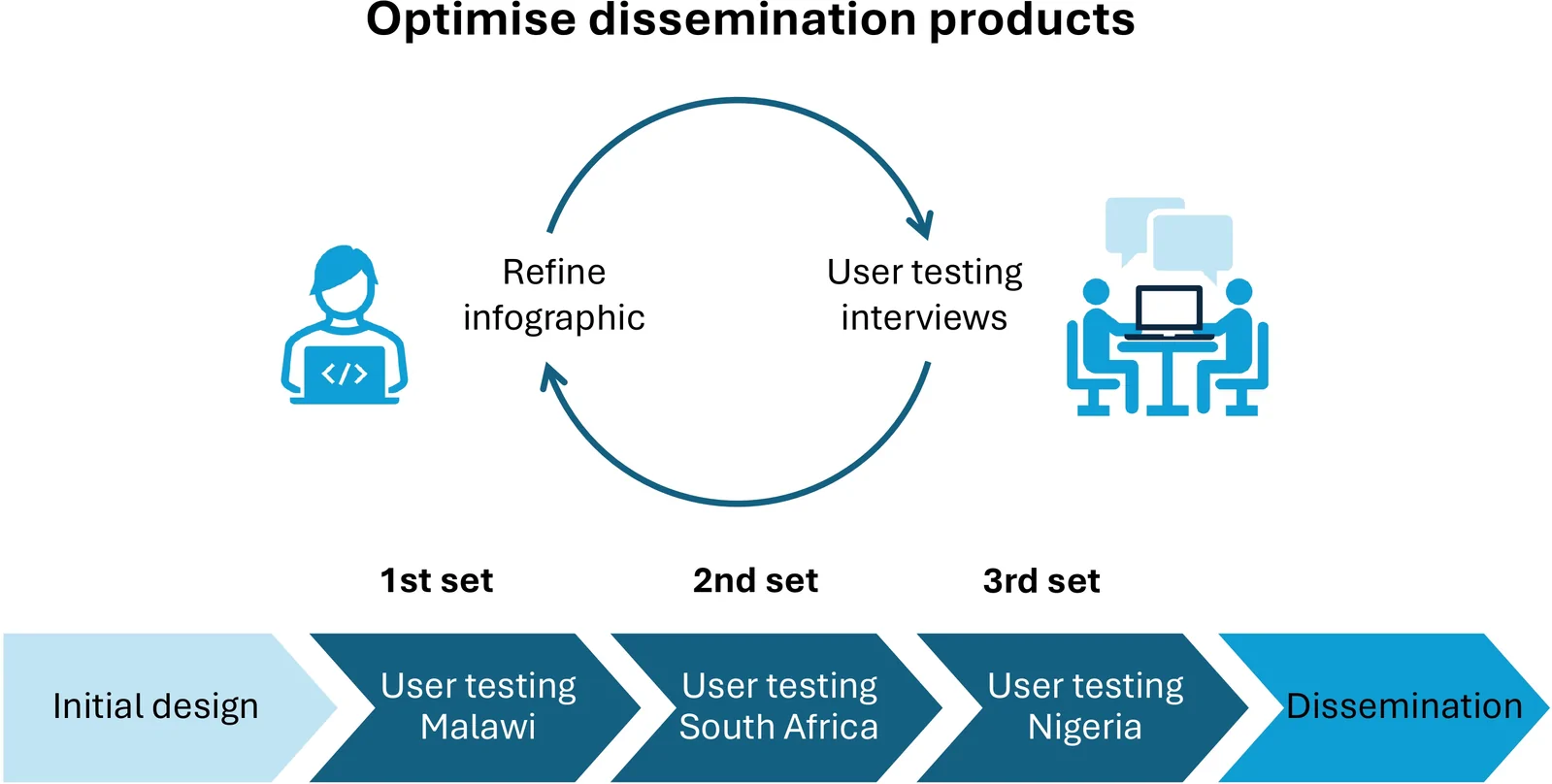
*:* Three sets of user testing interviews were conducted, with refinements to the infographics in between and at the end.

We decided to create different infographic versions for different audiences, including HCWs, policy makers and patients/guardians. This was based on an assumption that different audiences would likely differ with regard to the type of information needed and the type of language used. We also created landing pages to help direct users to the infographic version most appropriate for them. The user testing reported here only considered the versions for HCWs. Figures 3 and 4 show examples of the landing pages and infographics tested.

**Figure 3:**
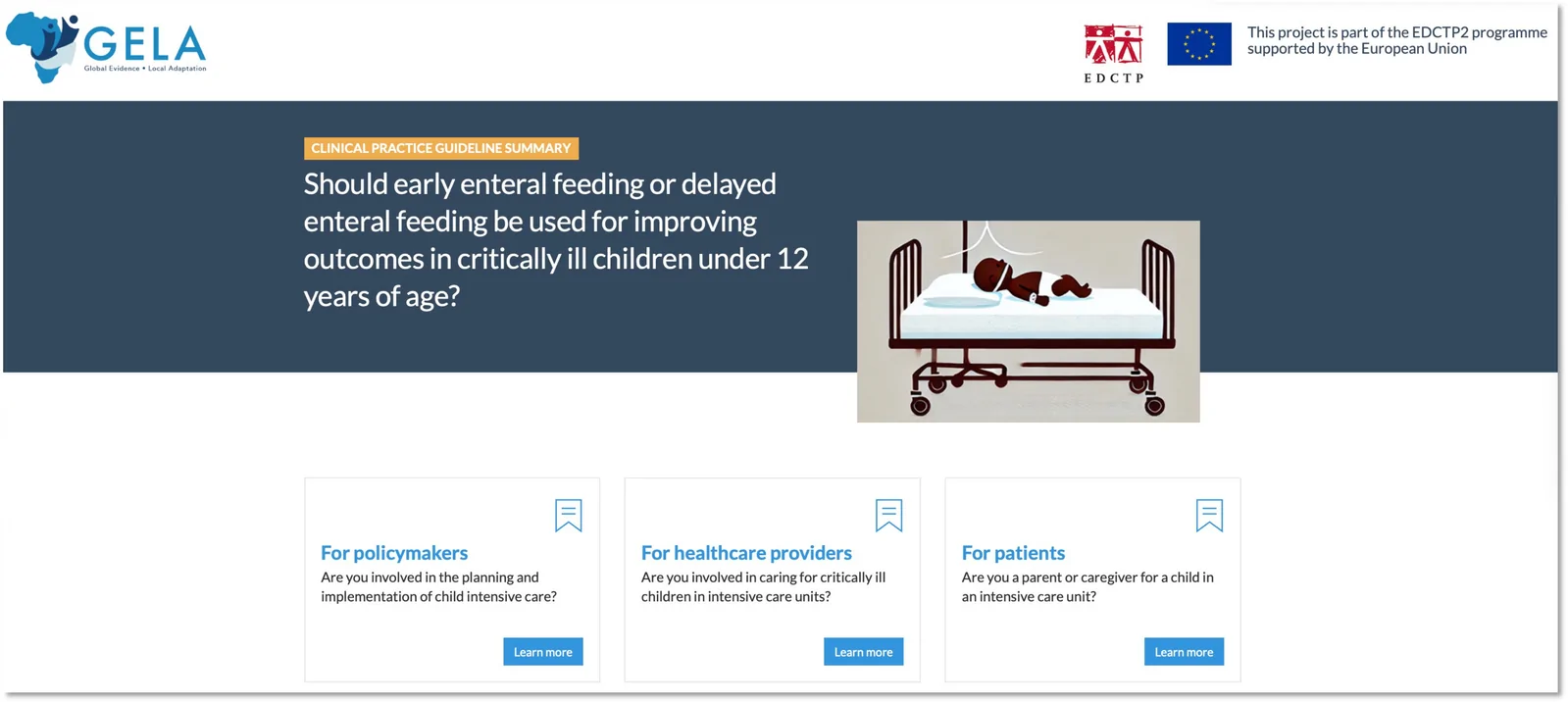
Example of landing page for CPG summary presented to participants in Malawi. CPG, clinical practice guideline.

**Figure 4:**
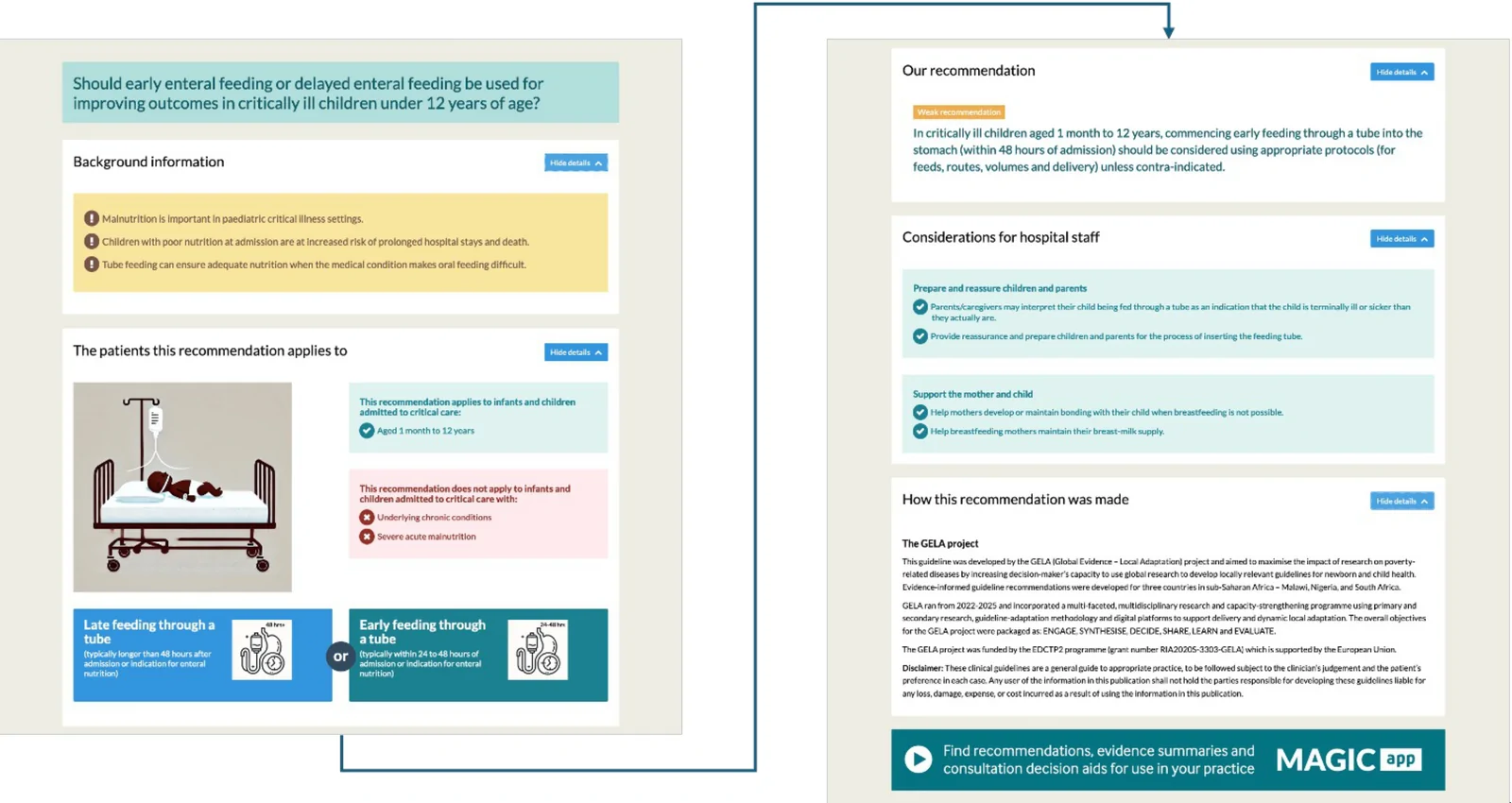
Example of CPG summary infographic presented to healthcare workers in Malawi. (For the purpose of illustration here, the infographic was cut in two parts placed side by side.) The final versions of the infographics for different audiences can be found here: https://portal.magicevidence.org/gela/*.* CPG, clinical practice guideline.

### Participants and setting

We conducted three sets of user testing interviews (see figure 2). We used purposive sampling to gather feedback from the expected target audience of the infographics, including participants with different roles in newborn and child care (eg, nurses, doctors, midwives, dieticians). The inclusion criterion was that the participants are practising HCWs within newborn or child health in Malawi, Nigeria or South Africa. Affiliation or participation in GELA was an exclusion criterion. Within the prescheduled number of interviews in each country, feedback on the infographics became repetitive, indicating practical saturation. Participants were identified by members of the GELA guideline development groups in each country and contacted by email. Interviews were voluntary with no additional incentive (participants provided written consent, see consent forms in online supplemental file 2). At all locations, the invited HCWs who found time in their schedule to meet us were generally eager to contribute to the study. All planned interviews were conducted, and none of the participants withdrew from the study.

The first set of interviews was conducted in Malawi, with HCWs from different units related to newborn and child health in the Kamuzu Central Hospital, Malawi College of Health Sciences Hospital, Lilongwe. The second set of interviews was conducted in South Africa, with HCWs from the Tygerberg University Hospital and the New Somerset Hospital in Cape Town, as well as a participant from the Children’s Institute at Cape Town University. The third set of interviews was conducted in Nigeria, with HCWs at the University of Calabar Teaching Hospital, Calabar. All participants were either involved in the care of children or newborns or in the teaching and administration of such care.

### Data collection

Participants met individually with the principal interviewer (TLH) and one local observer (SB in Malawi, WO or IIK in South Africa and MC or DA in Nigeria). An additional observer from Making GRADE the Irresistible Choice (VR) participated in the Malawi interviews. The principal interviewer and interview observers all have a background in public health research and guideline methodology, and they had received training in user testing from an experienced team member (CG).

The interviews took place during the period 14 October 2024–17 January 2025 at the workplace of the participant. All interviews were conducted in English, and no translation was used. Most interviews lasted for 1 hour, although some had to be shortened to 30–45 min due to participants’ availability. The dissemination product being tested was displayed on a laptop. The laptop was set up with OBS Studio (Open Broadcaster Software) to record the interview (audio and video) and capture the screen to see how participants navigated the infographics on the computer. The recordings were later transcribed. The observers also took notes during the interviews to help the analysis. One interview in Malawi was conducted with a printout version of the infographic, due to technical issues.

The interviews were conducted based on a semistructured interview guide (see online supplemental file 3). The initial ‘ice-breaker’ questions revolved around the participants’ professional roles and how they use and prefer to access CPGs in their workplace. The main part of the interviews included open and guided exploration of CPG summary infographics and specific questions related to the facets of user experience listed in table 2. At the end, participants were asked to share overall impressions and suggestions that could help improve either the infographics or the user testing interviews. The interview guide was reviewed by researchers from the GELA local partners in each country, and small adjustments were made between the interview rounds based on experience from previous rounds. During the interviews, participants were encouraged to ‘think aloud’ while looking at and exploring the dissemination products.

**Table 2:** Facets of user experience, adapted from Morville’s honeycomb model^24^

Facet	Explanation
Findability	Is it easy for users to navigate to the infographic and find the information they need?
Usability	Is it easy to use the infographic?
Understandability	Do users comprehend the language and contents of the infographic?
Desirability	Is the design of the infographic appealing to the user?
Identification	Can users relate to the contents (incl. images) of the infographic?
Accessibility	Is the infographic accessible and suitable for a broad range of target users, including users with special needs?
Credibility	Do the users find the information in the infographic trustworthy?
Usefulness	Does the infographic fill an information need for the user?
Value	Does the infographic deliver value to the end user in the sense that it can impact on care?

We acknowledge that the user testing setting may have introduced a Hawthorne effect,^23^ as participants knew they were being observed, and their engagement with the materials may differ from how they would use them during routine clinical practice. To address this, participants were encouraged to voice their honest opinions and ‘think aloud’, and it was emphasised at the beginning that they were not being tested and that no answer is wrong.

### Data analysis

We conducted qualitative content analysis using an adapted version of Morville’s honeycomb model^24^ (see table 2) while allowing inductive codes to emerge. Within each category, we then coded showstoppers (preventing further use), major frustration (hindering further use, with participants eventually figuring out the issue), minor frustration, positive feedback and suggestions for improvement of the infographic.^21^ Coding was conducted within each country to inform country-specific revisions to the infographics and later synthesised across countries to identify common issues. We did not conduct follow-up sessions with participants to discuss or confirm our interpretation of the findings.

The interviews were transcribed using the artificial intelligence (AI) tool Whisper Transcription V.10.3 for Mac. The principal interviewer (TLH), as well as one observer from each interview (VR, SB, WO, IIK, MC or DA), also listened to all recordings and verified the transcriptions. Observer notes were used to supplement the video and audio recordings. All identifiable features were removed. Transcriptions were then coded by the principal interviewer and validated by the observers. The validation of transcripts and coding by observers served to mitigate potential biases stemming from TLH co-designing the infographics and conducting the interviews. We used the Dedoose cloud application for managing, analysing and presenting qualitative and mixed-method research data (Los Angeles, California: SocioCultural Research Consultants, LLC www.dedoose.com).

## Results

### Participants

Table 3 presents the characteristics of the 26 interview participants, including 11 men and 15 women. Paediatricians were the most common profession (n=8), followed by researchers/lecturers (n=6) and nurses (n=4). Age is not reported because many participants preferred not to disclose it.

**Table 3:** Participant characteristics

Participant characteristics	Malawi (n=8)	South Africa (n=8)	Nigeria (n=10)
Gender	5 men 3 women	8 women	6 men 4 women
Profession	3 paediatricians 2 nurses 1 paediatric resident 1 lecturer 1 paediatric associate/clinical professor	3 paediatricians 1 neonatologist 1 lecturer 1 operational manager neonatal care 1 neonatal programme manager 1 communication and education specialist on child health	2 paediatricians 2 nurses 1 paediatric gastroenterologist 1 infection prevention and control consultant physician 1 midwife 1 dietician 1 lecturer/researcher 1 professor and hospital administrator

All but two participants reported using CPGs in their work: one did not work directly with patients but had encountered CPGs; the other had used CPGs previously and was involved in developing CPGs. Participants generally consulted CPGs occasionally, mainly to check unfamiliar or non-routine situations, verify information or identify new recommendations. Across all three countries, participants used national CPGs, and CPGs incorporated in hospital manuals and standard operating procedures. They also used Google and international online sources like Cochrane; UpToDate; Medline; Medscape; WHO; Centers for Disease Control and Prevention; American Academy of Pediatrics; European Society for Paediatric Gastroenterology, Hepatology and Nutrition; National Institute for Health and Care Excellence; and AMBOSS. Lack of contextualisation was mentioned as a reason for not using international CPGs.

### Users’ experience with the tested infographics

Table 4 summarises positive and negative feedback and suggestions for improvement of the infographics based on the 26 user testing interviews in the three countries. Below, we detail the findings for each facet, illustrated with selected quotes.

**Table 4:** Summary of key findings

Facet	Positive feedback	Negative feedback	Improvement suggestions
Findability		**Showstopper**: some were not able to navigate from the landing page and find more details in the infographic or link to MAGICapp **Major issue/frustration:** many overlooked information under ‘show details’ and did not find the link to the full infographic in MAGICapp (ie, did not recognise banner as a link to guideline)	Some suggested reordering the sections and making the recommendation more prominent at the top
Usability	Ability to click for more details was appreciated	**Major issue/frustration**: some struggled to navigate by clicking buttons	One suggested to fade out hidden content rather than hiding it completely One suggested to make the ‘Show more’ buttons blink One suggested to add a short video to explain how to use the infographic
Understandability	Simple language was appreciated	**Minor issue/frustration:** some raised concern about medicalised language **Major issue/frustration:** many were confused about who the recommendations applied to and the recommendation strength	
Desirability	Simple design, clean looks and pleasing colour palette were praised Many liked the pictures, which was the first thing that caught their eyes	**Minor issue/frustration:** some were concerned that the ‘Weak recommendation’ could make people doubt before they even read the recommendation **Minor issue/frustration:** some disliked the titles as questions as it can give the impression of uncertainty **Minor issue/frustration:** some said it is too much to read	
Identification	The contextualisation to their country made some participants identify more	**Minor issue/frustration:** some noted issues with images not accurately reflecting their work environment/setting	
Accessibility	Use non-serif font type was praised by one participant, as it increases readability	**Minor issue/frustration**: several participants commented about the font size being too small **Minor issue/frustration:** some questioned if it works on smartphones	Some recommended ways to share the infographics, including online platforms, QR codes in hospitals, social media and academic institutions
Credibility	Alignment with prior knowledge, explanations of how recommendations were made and link to of evidence created trust Professional and medically sound presentation and official branding gave credibility, and the mention of Malawi, Nigeria and South Africa was reassuring		Some suggested adding more details about the guideline developers and highlighting practitioner involvement
Usefulness	Sections with background information and implementation considerations and advice were mentioned as particularly useful		Some suggested adding more details, such as quantification of the problems, more practical guidance and links to other resources
Value	Many said infographics would be valuable in their work setting	**Major issue/frustration:** some said that infographics offered no new information and was more suited for people unfamiliar with the topic	
Other			Some suggestions went beyond the scope of the guidelines, such as changing the groups of patients the recommendations apply to

QR code, Quick-response code.

#### Findability

Participants were first shown a landing page with links to infographics for different audiences. Several participants in Malawi did not click the ‘Learn more’ buttons to access the infographics, so these were changed to ‘Click here’ before testing in South Africa and Nigeria. All South African participants then navigated successfully, but a few participants in Nigeria still struggled to move beyond the landing page. Some participants commented positively about the clear division by audience, but one suggested labelling the infographic versions and adding navigation buttons to return to the landing page, which we did in the final versions.

Inside the infographics, some participants overlooked the hidden sections under ‘Show details’, or noted that it might not be obvious for all users to click.

Several participants suggested placing the recommendations more prominently at the top:

I think that’s the first thing you should read when you come onto this page is this recommendation. Everything else is creating further clarity or qualifying that initial statement*.* (female South African communication and education specialist on child health)

If I don’t read anything else, you want me to read the recommendation. * (female South African neonatal programme manager)

The recommendations were therefore moved to the top in the final versions.

Many participants did not recognise the MAGICapp banner at the bottom of the infographic as a link to the full guideline, and some interpreted it as a video or advertising. The banner was consequently replaced with a text link: ‘Click here to see the full guideline and background evidence for the recommendation’.

#### Usability

Participants across Malawi, South Africa and Nigeria generally found the infographics user-friendly. As one participant noted:

A lot of people don’t want to read that much. So, this presentation, it’s clear, straight to the point on what you should do. And I like that it gives you an option to look at the full guideline in case you have any concerns or anything in it to follow up. (female Malawian paediatric resident)

While some participants appreciated being able to click for more details, some warned that it could discourage engagement:

I’ve heard from people, the more you have to click, the less people click. […] Have it open, have your image, have the recommendation and the key things. Then if you are wanting more detail, they can click for more details. (female South African neonatal programme manager)

#### Understandability

Most participants found the language and information easy to understand, describing the infographics as simple, concise and presented in ‘bite-sized chunks’. However, several participants suggested avoiding medical or academic terms such as ‘multi-component intervention’ and long sentences that could discourage people from reading all the information. We therefore simplified the language in the final versions.

Many participants across countries were unclear about which patient groups the recommendations applied to and what ‘usual care, no intervention’ meant. Participants sometimes interpreted the exclusion of a group as a recommendation against the intervention for that group. Many did not understand the graphics illustrating the intervention versus comparator. We therefore revised the wording describing eligible patient groups and removed the intervention/comparator graphics.

The recommendation strength labels (ie, ‘Weak recommendation’ or ‘Strong recommendation’) caused confusion, particularly in Malawi and South Africa. Some misunderstood what ‘weak recommendation’ referred to or questioned the need for these labels. We retained the labels in the final infographics, but added explanations of what they mean in terms of the balance of benefits and harms and whether most informed people would choose the recommended option.

#### Desirability

Participants generally praised the infographics’ clean design and appealing colours. One noted:

It looks interesting and somebody will want to pick it up and say: What’s it talking about? (female Nigerian paediatric gastroenterologist)

Many liked the images, which were often the first thing they noticed, but some suggested making them more prominent or using bolder colours, especially for printed versions.

It gave me the context which I liked in the image. It had a nice feel. The image I thought were reflected both nurturing and a professional feel. (female South African neonatal programme manager)

Some participants said the infographics contained too much text and that emphasising a ‘weak’ recommendation could reduce interest in the content. Others were concerned that question-based titles implied uncertainty, and preferred clear, actionable statements tailored to the target audience. We therefore shortened the contents and changed the headings from questions to statements in the final versions of the infographics.

#### Identification

Almost all participants were familiar with the infographic topics and could relate to the information contained in them because it was contextualised to their country. However, several participants commented that the images could be misleading or did not adequately represent the context. For example, some questioned the absence of caregivers in the Malawi enteral feeding infographic (figures 3 and 4), and one noted that the person with a stethoscope gave the impression that doctors conduct home visits in South Africa. Similarly, someone commented that the image in the Nigeria hand hygiene infographic failed to illustrate the interventions and contained distracting symbols, while another reacted to the many anxious-looking bystanders in the Nigeria enteral feeding infographic. We revised the imagery in the final versions in response to this feedback.

#### Accessibility

Some raised concerns that people unfamiliar with technology might struggle to navigate the digital infographics, especially on phones. Suggestions included fading out hidden content instead of hiding it completely, and adding a short video to explain how to use the infographic.

Participants in all three countries noted that some text was too small, but one commented that the non-serif font type was easy to read. We therefore retained the font type but increased the size in the final versions.

Participants emphasised that electronic versions should be simple, free and compatible with various devices, and possibly integrated with other hospital systems. Printed versions were also considered important where internet or device access is limited. Suggested dissemination channels included hospital websites, apps, QR codes in wards, social media, and academic and training institutions.

#### Credibility

Participants generally trusted the infographics because they aligned with their existing knowledge, and provided access to supporting evidence:

The fact that you could navigate to validate the evidence means it’s trustworthy*.* (male Nigerian professor and hospital administrator)

Other participants highlighted the professional and medically sound format, the official branding and the links to credible institutions instilled trust. Country-specific contextualisation was mentioned as reassuring, given concerns about the relevance of international CPGs:

Seeing Malawi and Nigeria, and South Africa kind of reassures me that this might relate to where I am. (male Malawian paediatric resident)

To improve the credibility further, one participant suggested adding information to show that practitioners had been involved in the CPG development.

#### Usefulness

Most participants found the infographics useful, noting that they provided clear, concise information while offering access to further details.

I think it’s pretty helpful. It’s clear. Straight to the point. If I need more details, then I’ll go to the MAGICapp, like you said. (male Malawian paediatric resident)

Participants appreciated the background information but often wanted more details, such as quantification of the problem, definitions of key terms and additional context. Advice to HCWs was similarly valued, although some participants requested more practical guidance or links to additional resources.

#### Value

Most participants in Malawi and Nigeria viewed the infographics as practically valuable. In Malawi, the enteral feeding infographic was seen as valuable for supporting evidence-based care when staff are unsure about procedures. In Nigeria, the hand hygiene infographic was valued for supporting infection control, while the enteral feeding infographic was seen as potentially valuable if integrated into hospital systems for automatic reminders and expanded to primary healthcare settings.

In contrast, some South African participants questioned the value of the home visits infographic, noting that it offered little new information and was more suited to those unfamiliar with home-based care:

There is no new information, and it seems more aimed towards someone who does not know what home-based care is. (female South African neonatologist)

It doesn’t give me enough information to act*.* (female South African communication and education specialist on child health)

## Discussion

### Principal findings

The user testing identified four key considerations for development of CPG summary infographics. First, the importance of clear visual hierarchy and prominent placement of recommendations. Second, the importance of using accessible language without losing the intended meaning. Third, to not only communicate what is recommended but also make the implications for practice readily accessible. Fourth, ensuring that illustrations are context appropriate, as visual elements can influence how users engage with and interpret the content. These findings provide practical insights for developing CPG dissemination products for HCWs in resource-constrained settings.

### Comparison with current literature

Previous studies have shown that user involvement in the design of CPG dissemination materials can help ensure that they are culturally, linguistically and visually appropriate, and suited to users’ health literacy levels and context.^25–28^ To our knowledge, this is the first study to user test CPG summary infographics in an low- and middle-income country setting with HCWs from diverse backgrounds and varying access to technology.

Our observations are consistent with previous research showing that HCWs and other users generally prefer infographics over text-based summaries, particularly for their visual appeal and reading experience.^11 29–31^ These findings support the use of infographics as a CPG dissemination format, while also highlighting the importance of tailoring materials to different audiences. This is consistent with GELA’s approach of developing separate versions for policy makers, HCWs and parents/guardians/patients, although further audience stratification may be needed.

Participants’ confusion about the recommendation strength labels also reflects findings from previous user testing studies of BMJ Rapid Recommendation infographics^13 14^ and the broader challenges of communicating evidence-based terminology.^32^ Such confusion and perceived uncertainty may impact CPG adherence.^33 34^

### Study limitations

The transferability of our findings is limited by the small number of infographics tested which focused on a narrow range of clinical topics, and by the user testing being confined to a few urban tertiary hospitals and academic settings, with no participation from rural areas. HCWs in rural settings may face different challenges and have different information needs from those represented in our sample.

Due to the difficulties in participant recruitment, we included a few participants who were already familiar with the GELA project, which may have introduced response bias or Hawthorne effect.^23^ We were unable to standardise interview durations because of time and resource constraints, leading to skipping immediate debriefings after some interviews. In addition, technical issues affected two interviews, requiring us to discard their audio/video recordings.

The involvement of TLH in both the design and user testing of infographics is a potential source of bias. However, her involvement in the infographic design was not communicated to participants. Moreover, her ability to talk to end users directly instead of relying on feedback collected by others strengthened our ability to implement the feedback in the final revisions.

### Implications for further research and development

Our findings suggest that CPG summary infographics are a promising dissemination tool, but their value depends on stakeholder involvement and integration into broader dissemination strategies and existing systems. Developing user-friendly infographics adds to the workload of CPG panels, and access to design expertise and co-creation processes may be limited. Although AI tools may help reduce this burden, they can also introduce contextual mismatches. Further research is therefore needed to understand how AI can best support infographic development. There is also a need to explore dissemination channels that are accessible in resource-constrained settings, including smartphone-based messaging services that do not rely on stable internet access.

Although we developed infographics for different target audiences, we lacked the capacity to conduct user testing with policy makers or citizens/guardians/mothers. Future research could explore how the needs and preferences of these groups differ from those of HCWs and how dissemination products could be adapted accordingly.

Some participants suggested changing the content and applicability of recommendations or adding information not supported by the source guidelines for the infographics. While such feedback could not be incorporated when revising the GELA infographics, we note that broader involvement of end users during the development of recommendations might have been valuable.

### Conclusions

User testing of CPG summary infographics with HCWs in Malawi, South Africa and Nigeria demonstrated their potential as accessible and time-efficient tools, while also identifying practical considerations for their development. Based on our findings, CPG summary infographics should place the recommendations prominently at the beginning, use simple and concise language, include information that supports implementation and ensure that visual elements are appropriate to the local clinical and cultural context. More broadly, our findings suggest that user involvement is key to developing effective CPG dissemination products.

## Supplementary material

10.1136/bmjopen-2026-122446 online supplemental file 1

10.1136/bmjopen-2026-122446 online supplemental file 2

10.1136/bmjopen-2026-122446 online supplemental file 3

10.1136/bmjopen-2026-122446 online supplemental file 4

10.1136/bmjopen-2026-122446 online supplemental file 5

10.1136/bmjopen-2026-122446 online supplemental file 6

10.1136/bmjopen-2026-122446 online supplemental file 7

## Data Availability

Data are available upon reasonable request.
