# Supplementary material for: Optimising summary infographics for clinical practice guidelines on child health: qualitative user testing study with healthcare workers in Malawi, Nigeria and South Africa
Source: BMJ Open. 2026 Oct 5;16(10):e122446. doi: 10.1136/bmjopen-2026-122446 (PMC13646941; doi:10.1136/bmjopen-2026-122446)
Supplement: Supplementary file 1 [file bmjopen-16-10-s004.pdf]

GELA
Global Evidence • Local Adaptation
EDCTP
This project is part of the EDCTP2 programme supported by the European Union

Should early enteral feeding or delayed enteral feeding be used for improving outcomes in critically ill children under 12 years of age?

Background information

- Malnutrition is important in paediatric critical illness settings.
- Children with poor nutrition at admission are at increased risk of prolonged hospital stays and death.
- Tube feeding can ensure adequate nutrition when the medical condition makes oral feeding difficult.

The patients this recommendation applies to

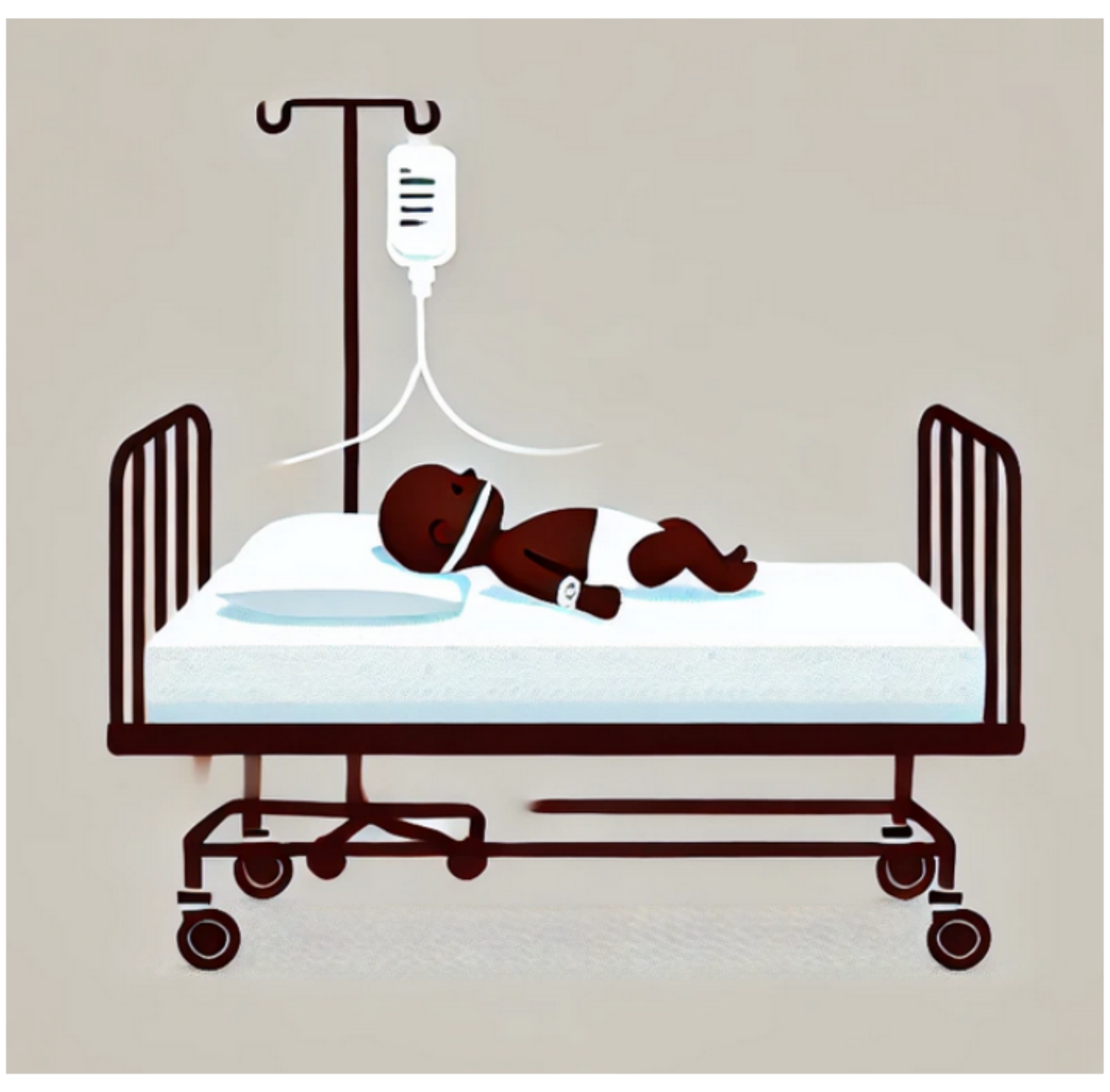

This recommendation applies to infants and children admitted to critical care:

- Aged 1 month to 12 years

This recommendation does not apply to infants and children admitted to critical care with:

- Underlying chronic conditions
- Severe acute malnutrition

Late feeding through a tube
(typically longer than 48 hours after admission or indication for enteral nutrition)

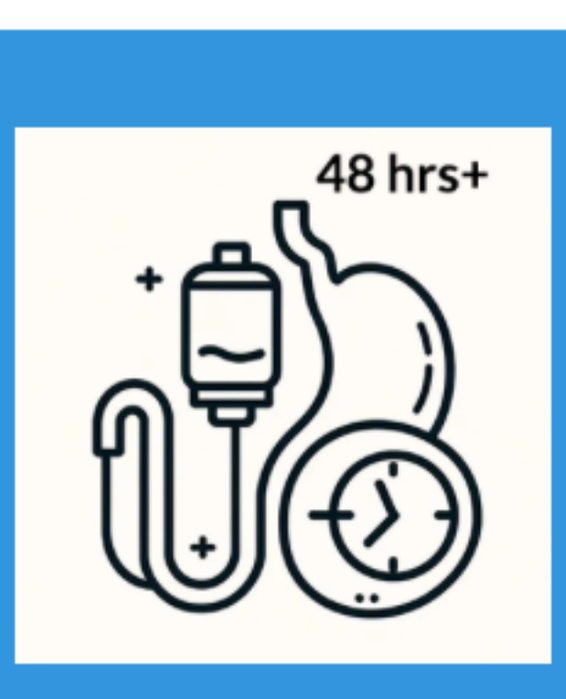

or

Early feeding through a tube
(typically within 24 to 48 hours of admission or indication for enteral nutrition)

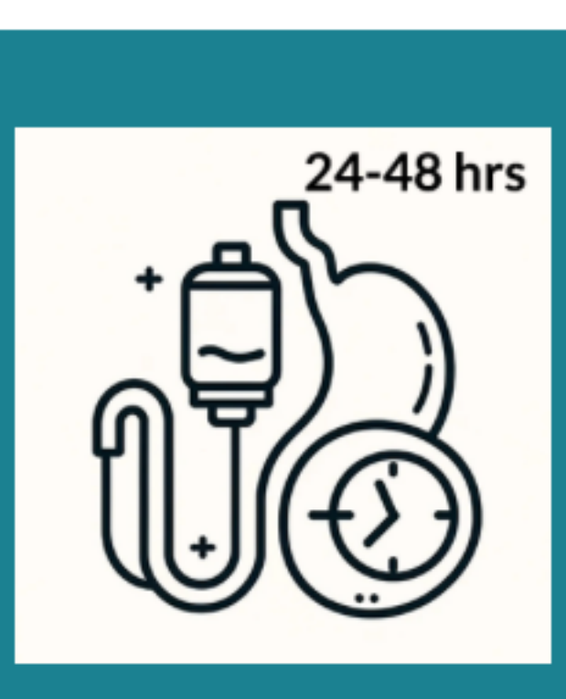

Our recommendation

Weak recommendation

Benefits outweigh harms for the majority, but not for everyone. The majority of patients would likely want this option.

In critically ill children aged 1 month to 12 years, commencing early feeding through a tube into the stomach (within 48 hours of admission) should be considered using appropriate protocols (for feeds, routes, volumes and delivery) unless contra-indicated.

Considerations for hospital staff

Prepare and reassure children and parents

- Parents/caregivers may interpret their child being fed through a tube as an indication that the child is terminally ill or sicker than they actually are.
- Provide reassurance and prepare children and parents for the process of inserting the feeding tube.

Support the mother and child

- Help mothers develop or maintain bonding with their child when breastfeeding is not possible.
- Help breastfeeding mothers maintain their breast-milk supply.

How this recommendation was made

The GELA project

This guideline was developed by the GELA (Global Evidence – Local Adaptation) project and aimed to maximise the impact of research on poverty-related diseases by increasing decision-maker's capacity to use global research to develop locally relevant guidelines for newborn and child health. Evidence-informed guideline recommendations were developed for three countries in sub-Saharan Africa – Malawi, Nigeria, and South Africa.

GELA ran from 2022-2025 and incorporated a multi-faceted, multidisciplinary research and capacity-strengthening programme using primary and secondary research, guideline-adaptation methodology and digital platforms to support delivery and dynamic local adaptation. The overall objectives for the GELA project were packaged as: ENGAGE, SYNTHESISE, DECIDE, SHARE, LEARN and EVALUATE.

The GELA project was funded by the EDCTP2 programme (grant number RIA2020S-3303-GELA) which is supported by the European Union.

Disclaimer: These clinical guidelines are a general guide to appropriate practice, to be followed subject to the clinician's judgement and the patient's preference in each case. Any user of the information in this publication shall not hold the parties responsible for developing these guidelines liable for any loss, damage, expense, or cost incurred as a result of using the information in this publication.

Find recommendations, evidence summaries and consultation decision aids for use in your practice
MAGIC app
