# Supplementary material for: Optimising summary infographics for clinical practice guidelines on child health: qualitative user testing study with healthcare workers in Malawi, Nigeria and South Africa
Source: BMJ Open. 2026 Oct 5;16(10):e122446. doi: 10.1136/bmjopen-2026-122446 (PMC13646941; doi:10.1136/bmjopen-2026-122446)
Supplement: Supplementary file 2 [file bmjopen-2026-122446.reviewer_comments.pdf]

PEER REVIEW HISTORY

BMJ Open publishes all reviews undertaken for accepted manuscripts. Reviewers are asked to complete a checklist review form and are provided with free text boxes to elaborate on their assessment. These free text comments are reproduced below.

ARTICLE DETAILS

Title (Provisional)

Optimizing summary infographics for clinical practice guidelines on child health: qualitative user testing study with healthcare workers in Malawi, Nigeria and South Africa

Authors

Lubelwana Hafver, Tandekile; Achille, Frankie; Reinthaler, Valerie; Nagraj, Sumanth; Banda, Susan; Odendaal, Willem; Kallon, Idriss I; Chibuzor, Moriam; Arikpo, Dachi; Chipojola, Roselyn; Effa, Emmanuel; Mbeye, Nyanyiwe Masingi; Kredo, Tamara; Leong, Trudy Desirie D; Lewin, Simon; Brand, Amanda Salomé; Glenton, Claire; Cooper, Sara; Schmidt, Bey-Marrié; Kyomuhendo Munabi-Babigumira, Susan; Vandvik, Per O.; Agoritsas, Thomas; Delvaux , Nicolas

---

VERSION 1 - REVIEW

**Reviewer**	**1**
**Name**	**Mbalinda, Scovia**
**Affiliation**	**Makerere University, Nursing**
**Date**	**21-Jul-2026**
**COI**	**None**

Overall assessment

- The study addresses an important and underexplored aspect of guideline implementation in LMICs: how clinical practice guideline (CPG) infographics are understood and used by healthcare workers in Malawi, Nigeria, and South Africa.
- The qualitative user-testing design, multi-country scope, and explicit use of SRQR are appropriate and clearly described, but the manuscript is long, somewhat repetitive, and the main messages could be more sharply articulated for BMJ Open's broad readership.

Please see detailed comments per section below.

Title and abstract

- The title is informative and clearly identifies the design ("qualitative user testing study") and target population (HCWs in three African countries), which is consistent with BMJ Open

guidance.

- The abstract is comprehensive but almost reads like a mini-paper, with several long sentences and some repetition of background material. Key problems (e.g., confusion around recommendation strength, difficulty with navigation, and variable perceived usefulness) are mentioned but could be more explicitly linked to "what changed" in the infographics and "what this means" for practice and future dissemination work. FOR EXAMPLE

"Key problems … could be more explicitly linked to 'what changed'"

Right now, the abstract mentions issues such as:

- Confusion around recommendation strength.
- Difficulties with navigation/layout.
- Variable perceived usefulness across topics and countries.

But it doesn't clearly say:

- How these problems led you to adjust the infographics.
- What design decisions you made as a result.
- What your findings imply for future guideline dissemination work.

Readers will want to see:

- "HCWs struggled with X, so we did Y to the infographics, which suggests Z for others designing guideline tools."

Concretely, you could restructure the Results and Conclusions part of the abstract like this:

- Results:
  - "HCWs appreciated implementation sections but struggled to interpret recommendation strength and target populations."
  - "Navigation and imagery were sometimes misaligned with local contexts."
- Conclusions/Implications:
  - "We clarified recommendation strength, re-organised layouts to foreground actionable content, and refined imagery. Future guideline projects should integrate user testing early and design smartphone-friendly, locally contextualised infographics."

That way, the abstract doesn't just list problems; it shows:

1. What went wrong.
2. What you changed.
3. What this means for practice and future dissemination work.

Introduction

- The introduction appropriately frames CPGs as part of an "evidence ecosystem" and positions infographics as dissemination tools, with references to prior user-testing work in Europe and elsewhere.

- The rationale for focusing on newborn and child health within the GELA project and for targeting HCWs in Malawi, Nigeria, and South Africa is clear but could be more concise; some paragraphs repeat ideas about accessibility/usability of guidelines and evidence ecosystems.

Suggestions

- Condense the background:
  - One paragraph on the quality gap, evidence ecosystem, and CPGs.
  - One paragraph on infographics and the gaps in existing research (e.g., limited evidence from LMICs, limited exploration of user experience among HCWs).
- Strengthen the problem statement: explicitly state what is unknown (e.g., "How do HCWs in LMIC settings experience CPG infographics, and what design features facilitate or hinder use?").
- State the aims more explicitly (e.g., "to explore HCWs' preferences; to identify barriers and facilitators; to inform refinement of infographics and future guideline dissemination strategies").

Methods

Design and setting

- The user-testing design is described clearly, and the link between the GELA guideline development work and this dissemination-focused work package is well presented (Figure 1 and Figure 2 are helpful).
- The use of SRQR and a specific user-test toolkit, plus multi-country purposive sampling, aligns well with BMJ Open expectations for transparent qualitative reporting.

Suggestions

- Consider moving some of the very detailed SRQR checklist text and the full interview guide to supplementary material (if not already) and keep the main Methods section more concise, referring to "see Supplementary File X for full SRQR mapping and interview guide."
- Clarify the qualitative approach (e.g., "descriptive qualitative study using think-aloud user-testing interviews, informed by Morville's user experience honeycomb") rather than only referencing SRQR and toolkits; readers appreciate an explicit statement of the qualitative approach/paradigm.

Participants and sampling

- Participant recruitment through GELA guideline development group members and purposive sampling to capture different roles (nurses, doctors, midwives, dieticians, lecturers, etc.) are well-described, and Table 3 nicely summarizes participant characteristics.
- The concept of "practical saturation" is mentioned but not fully explained in terms of how it was assessed (e.g., specific signals in the data that suggested saturation).

Suggestions

- Add a short explanation of how saturation was judged (e.g., "we considered saturation

reached when no new user-experience issues were identified in two consecutive interviews within each country”).

- Clarify whether any participants declined or withdrew and whether there were differences in engagement between roles or sites, even if brief (this helps readers understand potential selection bias).

Data collection

- The use of OBS Studio to record screen and audio, plus local observers and note-taking, provides rich data and demonstrates methodological rigor.
- The semi-structured interview guide is extensive and clearly linked to Morville’s honeycomb facets (findability, usability, understandability, desirability, identification, accessibility, credibility, usefulness, value).

Suggestions

- The manuscript mentions the use of a semi-structured interview guide (see Supplementary File 3) but does not detail its structure in the main text. I suggest including a brief overview in the Data collection section that describes the three main parts of the guide: (1) initial 'ice-breaker' questions about participants' roles and current guideline use; (2) open-ended exploration where participants think aloud while engaging with the infographic; and (3) guided exploration based on Morville's user-experience honeycomb, covering aspects like findability, usability, understandability, desirability, identification, accessibility, credibility, usefulness, and value. This addition would help readers understand the interview process at a glance without needing to consult the supplementary file, which can still contain the full guide.
- Clarify whether interviews were conducted in English or local languages and how language choice may have influenced discussions about medical jargon and recommendation strength.
- Indicate how potential “Hawthorne effect” influences were handled or acknowledged, beyond just citing the concept in the Discussion.

Data processing and analysis

- The description of transcription, coding, and verification (local observers checking transcripts and coding) suggests careful handling of data and attempts to enhance trustworthiness.
- The analytic approach (identifying themes across honeycomb facets and across countries) is implied but could be more explicitly named (e.g., “framework analysis using Morville’s facets as an analytical scaffold”) and linked to the production of Table 4.

Suggestions

- Explicitly state the analytic method (e.g., “We conducted qualitative content analysis using Morville’s facets as a deductive framework, while allowing inductive codes to emerge; coding was conducted by XX and checked by YY, with disagreements resolved through discussion”).
- Add a brief description of how cross-country comparisons were handled (e.g., matrix coding, separate within-country analyses then synthesis).
- Describe techniques to enhance trustworthiness more concretely:
    - Use of multiple coders and observer notes.

    - Any reflexivity/positionality considerations, especially given that some authors co-designed the infographics and conducted interviews.
    - Whether any member checking or feedback sessions occurred.

Results

Structure and clarity

- The Results are well organized around user experience facets (Findability, Usability, Understandability, Desirability, Identification, Accessibility, Credibility, Usefulness, Value) and supported by a concise summary table (Table 4) listing positive feedback, negative feedback, and improvement suggestions.
- The multi-country context is presented, but country-specific nuances could be made more explicit; currently, much of the narrative reads as aggregated findings with only occasional references to differences by site or topic.

Strengths

- Clear identification of key issues:
    - Difficulty navigating landing pages and recognizing links to MAGICapp.
    - Confusion about recommendation strength (weak vs strong) and target populations/intervention/comparator distinctions.
    - Appreciation of implementation considerations and clear background sections.
    - Mixed reactions to imagery, particularly when not reflective of local contexts.
- Use of Morville's model provides a structured lens that is likely to be useful for guideline developers and digital product designers.

Suggestions

- Include more direct quotes (in the main text rather than only implied) to illustrate the most important points for each facet, while keeping the number manageable; Readers expects illustrative quotations to substantiate thematic claims.
- Make country or topic differences more explicit: for example, did HCWs dealing with infection prevention and control vs enteral feeding or family-centered support perceive the infographics differently?
- Clarify which suggestions were acted upon in the final infographics and which remained aspirational; this would help readers see the "impact" of user testing on design.

Discussion

Interpretation and linkage to prior work

- The Discussion appropriately situates findings in relation to previous work on infographics, visual abstracts, and guideline dissemination, including European user-testing studies and participatory design literature.
- The authors highlight the challenge of explaining evidence-based concepts (e.g., GRADE recommendation strength) to both HCWs and lay audiences, and the tension between simplifying language and preserving scientific accuracy.

Suggestions

- Shorten and sharpen the Discussion:
  - Begin with a clear statement of the 3–4 most important findings (e.g., navigation issues, confusion about recommendation strength and target populations, value of implementation sections, importance of locally contextualized imagery).
  - Then discuss how these findings confirm, extend, or challenge previous studies (e.g., BMJ Rapid Recommendations user-testing in Europe).
- Elaborate more explicitly on how these findings might generalize to other guideline projects in LMICs (including maternal and newborn health), not only child health, while acknowledging contextual limits.
- Reflect more deeply on the implications of having some authors both design the infographics and conduct user testing: how might this influence participant feedback or interpretation, and how was this mitigated?

Strengths and limitations

- The "Strengths and limitations" box is clear and appropriately located; it notes the think-aloud approach, multi-country sample, and topic diversity, as well as the small sample and limited transferability.
- Limitations related to potential social desirability bias, Hawthorne effect, and selection bias (participants recruited via guideline groups) are noted but could be briefly elaborated.

Suggestions

- Clearly state that most participants were healthcare workers involved in tertiary hospitals and academic settings; explain that this might restrict the relevance of findings to district-level facilities or community health workers.
- Include a limitation about using AI-generated images, noting that it could cause minor contextual mismatches and that future research should focus on more participatory image creation. With end users.

Conclusions and implications

- The conclusions correctly emphasise that CPG infographics can be useful quick-reference tools, but issues such as navigation, layout, recommendation visibility, and clarity about recommendation strength and target population require attention.
- There is a useful observation that stakeholder engagement should begin earlier in the guideline development process, not only during dissemination and product design.

Suggestions

- Make the conclusions more action-oriented:
  - Highlight 3–4 concrete design recommendations (e.g., "place key recommendations and target population information at the top; minimise jargon and clarify intervention/comparator; ensure imagery is co-designed with local HCWs; use smartphone-friendly formats and clear links to full guidelines").
- Consider adding a short paragraph on next steps: whether there are plans to evaluate real-

world use of the refined infographics in clinical practice, or to extend the approach to other guideline topics (including maternal and newborn care).

Specific BMJ Open style recommendations

1. **Patient and public involvement (PPI)**
   - You appropriately note that patients/public were not involved and justify this.
   - Consider adding a brief reflection on whether future work might benefit from including parents/caregivers in infographic design for patient-facing versions, even if beyond the current scope.
2. **Data sharing statement**
   - The current statement "datasets are available from the corresponding author on reasonable request" is acceptable, but BMJ Open often encourages more detail

(e.g., what type of data transcripts, coded data, analytic memos can be shared, and with what restrictions).

3. **Funding and competing interests**
   - The funding statement is clear and meets BMJ Open requirements; the role of EDCTP2 is appropriately described.
   - Competing interests are reported, but given that several authors co-designed the infographics and led the user testing, consider explicitly framing this as a "non-financial competing interest" and explaining how you attempted to mitigate bias.
4. **Strength of recommendations vs strength of evidence**
   - Since confusion around "weak" vs "strong" recommendations is central to your findings, consider inserting a short explanatory note in the Discussion to help readers unfamiliar with GRADE understand the implications.

VERSION 1 - AUTHOR RESPONSE

Dr. Scovia Mbalinda (Reviewer 1),

> ***Thank you for your thorough review and constructive feedback. Please see our replies to your comments below.***

Overall assessment
• The study addresses an important and underexplored aspect of guideline implementation in LMICs: how clinical practice guideline (CPG) infographics are understood and used by healthcare workers in Malawi, Nigeria, and South Africa.
• The qualitative user-testing design, multi-country scope, and explicit use of SRQR are appropriate and clearly described, but the manuscript is long, somewhat repetitive, and the main messages could be more sharply articulated for BMJ Open's broad readership.

> ***Thank you for this positive feedback. We have used your suggestions below to sharpen the messages. We have also shortened the "Background", "Results" and "Discussion" so that the new version stays within BMJ Open's word limit of 3-4000 words.***

Please see detailed comments per section below.

Title and abstract
• The title is informative and clearly identifies the design ("qualitative user testing study") and target population (HCWs in three African countries), which is consistent with BMJ Open guidance.
• The abstract is comprehensive but almost reads like a mini-paper, with several long sentences and some repetition of background material. Key problems (e.g., confusion around recommendation strength, difficulty with navigation, and variable perceived usefulness) are mentioned but could be more explicitly linked to "what changed" in the infographics and "what this means" for practice and future dissemination work. FOR EXAMPLE
"Key problems ... could be more explicitly linked to 'what changed'"
Right now, the abstract mentions issues such as:
• Confusion around recommendation strength.
• Difficulties with navigation/layout.
• Variable perceived usefulness across topics and countries.
But it doesn't clearly say:
• How these problems led you to adjust the infographics.
• What design decisions you made as a result.
• What your findings imply for future guideline dissemination work.
Readers will want to see:
• "HCWs struggled with X, so we did Y to the infographics, which suggests Z for others designing guideline tools."
Concretely, you could restructure the Results and Conclusions part of the abstract like this:
• Results:
o "HCWs appreciated implementation sections but struggled to interpret recommendation strength and target populations."
o "Navigation and imagery were sometimes misaligned with local contexts."
• Conclusions/Implications:
o "We clarified recommendation strength, re-organised layouts to foreground actionable content, and refined imagery. Future guideline projects should integrate user testing early and design smartphone friendly, locally contextualised infographics."
That way, the abstract doesn't just list problems; it shows:
1. What went wrong.
2. What you changed.
3. What this means for practice and future dissemination work.

> ***Thank you for this very helpful comment. We agree that the previous version of the abstract was missing how the feedback we received was used and has implications for future guideline development projects. We have updated the Results and Conclusions section inspired by your suggestions. In addition, we have replaced the Background and Methods sections with a new structure requested by the editor. We hope the new abstract is more informative to the reader.***

Introduction
• The introduction appropriately frames CPGs as part of an "evidence ecosystem" and positions infographics as dissemination tools, with references to prior user-testing work in Europe and elsewhere.
• The rationale for focusing on newborn and child health within the GELA project and for

targeting HCWs in Malawi, Nigeria, and South Africa is clear but could be more concise; some paragraphs repeat ideas about accessibility/usability of guidelines and evidence ecosystems.
Suggestions
• Condense the background:
o One paragraph on the quality gap, evidence ecosystem, and CPGs.
o One paragraph on infographics and the gaps in existing research (e.g., limited evidence from LMICs, limited exploration of user experience among HCWs).
• Strengthen the problem statement: explicitly state what is unknown (e.g., "How do HCWs in LMIC settings experience CPG infographics, and what design features facilitate or hinder use?").
• State the aims more explicitly (e.g., "to explore HCWs' preferences; to identify barriers and facilitators; to inform refinement of infographics and future guideline dissemination strategies").

Based on your advice to condense the Introduction and strengthen the problem statement and aims, we have made these changes in the "Background" section:

- ***We have removed the following sentence, because this information is repeated later in the discussion: "However, these studies also noted that users struggled with certain evidence-based medicine terminology and concepts, such as the Grading of Recommendations Assessment, Development and Evaluation (GRADE) framework[15]. "***
- ***Instead of the removed sentence, we end the paragraph with a statement of the research problem: "However, evidence is limited on how healthcare workers in resource-constrained settings experience and use such CPG summary infographics."***
- ***We changed and shortened the last paragraph to focus on the study aims: "As part of the he Global Evidence, Local Adaptation (GELA) project15, whose overarching goal was to strengthen decision makers' capacity to use global research evidence to develop locally contextualized CPGs, this study aimed to optimise CPG summary infographics on newborn and child health for use by HCWs in Malawi, Nigeria and South Africa. We conducted user-testing interviews with local HCWs to identify content and design features that facilitate or hinder use, informing refinement of the infographics before final dissemination."***

Methods
Design and setting
• The user-testing design is described clearly, and the link between the GELA guideline development work and this dissemination-focused work package is well presented (Figure 1 and Figure 2 are helpful).
• The use of SRQR and a specific user-test toolkit, plus multi-country purposive sampling, aligns well with BMJ Open expectations for transparent qualitative reporting.
Suggestions
• Consider moving some of the very detailed SRQR checklist text and the full interview guide to supplementary material (if not already) and keep the main Methods section more concise, referring to "see Supplementary File X for full SRQR mapping and interview guide."

• Clarify the qualitative approach (e.g., “descriptive qualitative study using think-aloud user-testing interviews, informed by Morville’s user experience honeycomb”) rather than only referencing SRQR and toolkits; readers appreciate an explicit statement of the qualitative approach/paradigm.

- ***The SRQR checklist is already in supplementary file 1 and not described in the main text.***
- ***We changed the second paragraph under “Methods”>>”Study design” to clarify that: “This is a descriptive qualitative study using think-aloud user-testing interviews, based on a user test toolkit…”***
- ***We decided to not mention Morville’s framework under “Study design”, because we think this information fits naturally where it is now under “Data analysis” and in table 2, and we prefer to avoid repetition.***

Participants and sampling
• Participant recruitment through GELA guideline development group members and purposive sampling to capture different roles (nurses, doctors, midwives, dieticians, lecturers, etc.) are well-described, and Table 3 nicely summarizes participant characteristics.
• The concept of “practical saturation” is mentioned but not fully explained in terms of how it was assessed (e.g., specific signals in the data that suggested saturation).
Suggestions
• Add a short explanation of how saturation was judged (e.g., “we considered saturation reached when no new user-experience issues were identified in two consecutive interviews within each country”).
• Clarify whether any participants declined or withdrew and whether there were differences in engagement between roles or sites, even if brief (this helps readers understand potential selection bias).

We thank you for your suggestions to clarify this section for the readers, and have made the following changes:

- ***We replaced the sentence “The focus was to gather actionable feedback to optimize the infographics, and sampling stopped when feedback became repetitive, indicating practical saturation” with “Within the prescheduled number of interviews in each country, feedback on the infographics became repetitive, indicating practical saturation.” This clarifies that we conducted the scheduled interviews and did not cancel any scheduled interviews because we felt that we already had enough feedback.***
- ***We added a sentence: “At all locations, the invited HCWs who found time in their schedule to meet us were generally eager to contribute to the study. All planned interviews were conducted, and none of the participants withdrew from the study.”***

Data collection
• The use of OBS Studio to record screen and audio, plus local observers and note-taking, provides rich data and demonstrates methodological rigor.
• The semi-structured interview guide is extensive and clearly linked to Morville’s honeycomb facets (findability, usability, understandability, desirability, identification, accessibility,

credibility, usefulness, value).
Suggestions
• The manuscript mentions the use of a semi-structured interview guide (see Supplementary File 3) but does not detail its structure in the main text. I suggest including a brief overview in the Data collection section that describes the three main parts of the guide: (1) initial 'ice-breaker' questions about participants' roles and current guideline use; (2) open-ended exploration where participants think aloud while engaging with the infographic; and (3) guided exploration based on Morville's user-experience honeycomb, covering aspects like findability, usability, understandability, desirability, identification, accessibility, credibility, usefulness, and value. This addition would help readers understand the interview process at a glance without needing to consult the supplementary file, which can still contain the full guide.
• Clarify whether interviews were conducted in English or local languages and how language choice may have influenced discussions about medical jargon and recommendation strength.
• Indicate how potential “Hawthorne effect” influences were handled or acknowledged, beyond just citing the concept in the Discussion.

We agree with your suggestions and have made the following adjustments:

- ***We added a sentence stating: “All interviews were conducted in English, and no translation was used.”***
- ***We added a short description of the interview contents in the main text: “The initial “ice-breaker” questions revolved around the participants’ professional roles and how they use and prefer to access CPGs in their workplace. The main part of the interviews included open and guided exploration of CPG summary infographics and specific questions related to the facets of user experience listed in Table 2. At the end, participants were asked to share overall impressions and suggestions that could help improve either the infographics or the user-testing interviews.”***
- ***Regarding potential Hawthorne effect, we added a paragraph to explain: “We acknowledge that the user-testing setting may have introduced a Hawthorne effect23, as participants knew they were being observed, and their engagement with the materials may differ from how they would use them during routine clinical practice. To address this, participants were encouraged to voice their honest opinions and “think aloud”, and it was emphasised at the beginning that they were not being tested, and that no answer is wrong.”***

Data processing and analysis
• The description of transcription, coding, and verification (local observers checking transcripts and coding) suggests careful handling of data and attempts to enhance trustworthiness.
• The analytic approach (identifying themes across honeycomb facets and across countries) is implied but could be more explicitly named (e.g., “framework analysis using Morville’s facets as an analytical scaffold”) and linked to the production of Table 4.
Suggestions
• Explicitly state the analytic method (e.g., “We conducted qualitative content analysis using Morville’s facets as a deductive framework, while allowing inductive codes to emerge; coding was conducted by XX and checked by YY, with disagreements resolved through discussion”).
• Add a brief description of how cross-country comparisons were handled (e.g., matrix coding,

separate within-country analyses then synthesis).
• Describe techniques to enhance trustworthiness more concretely:
o Use of multiple coders and observer notes.
o Any reflexivity/positionality considerations, especially given that some authors co-designed the infographics and conducted interviews.
o Whether any member checking or feedback sessions occurred.

To address your comments, we have made the following changes to the "Data analysis" section:

1) ***We swapped the order of the two paragraphs to highlight the data analysis approach first.***
2) ***We have been more explicit about our approach as suggested by you: "We conducted qualitative content analysis using an adapted version of Morville's honeycomb model24 (see Table 2) while allowing inductive codes to emerge."***
3) ***Regarding cross-country comparisons, we added this sentence: "Coding was conducted within each country to inform country-specific revisions to the infographics and later synthesised across countries to identify common issues."***
4) ***We added a sentence: "Observer notes were used to supplement the video and audio recordings."***
5) ***We modified a sentence to highlight how we managed potential bias : "The validation of transcripts and coding by observers served to mitigate potential biases stemming from TLH co-designing the infographics and conducting the interviews."***
6) ***Regarding member checking and feed-back sessions, we added a sentence: "We did not conduct follow-up sessions with participants to discuss or confirm our interpretation of the findings."***

Results
Structure and clarity
• The Results are well organized around user experience facets (Findability, Usability, Understandability, Desirability, Identification, Accessibility, Credibility, Usefulness, Value) and supported by a concise summary table (Table 4) listing positive feedback, negative feedback, and improvement suggestions.
• The multi-country context is presented, but country-specific nuances could be made more explicit; currently, much of the narrative reads as aggregated findings with only occasional references to differences by site or topic.
Strengths
• Clear identification of key issues:
o Difficulty navigating landing pages and recognizing links to MAGICapp.
o Confusion about recommendation strength (weak vs strong) and target populations/intervention/comparator distinctions.
o Appreciation of implementation considerations and clear background sections.
o Mixed reactions to imagery, particularly when not reflective of local contexts.
• Use of Morville's model provides a structured lens that is likely to be useful for guideline developers and digital product designers.
Suggestions

• Include more direct quotes (in the main text rather than only implied) to illustrate the most important points for each facet, while keeping the number manageable; Readers expects illustrative quotations to substantiate thematic claims.
• Make country or topic differences more explicit: for example, did HCWs dealing with infection prevention and control vs enteral feeding or family-centered support perceive the infographics differently?
• Clarify which suggestions were acted upon in the final infographics and which remained aspirational; this would help readers see the "impact" of user testing on design.

- ***We have shortened the results section to be more concise.***
- ***We decided to leave the quotes separate from the main text so that they stand out. Note that each quote is used to illustrate the points made in the main text immediately above it, and we have tried to make this clearer.***
- ***Regarding country differences, it should be noted that the participants in the different countries viewed different infographics on different topics. Therefore, we believe that a more explicit direct comparison between countries is difficult. Instead, we have tried to extract learnings of general value from all countries.***
- ***We have added/modified sentences under the different facets to indicate changes we made to the final versions based on the feedback we received.***

Discussion
Interpretation and linkage to prior work
• The Discussion appropriately situates findings in relation to previous work on infographics, visual abstracts, and guideline dissemination, including European user-testing studies and participatory design literature.
• The authors highlight the challenge of explaining evidence-based concepts (e.g., GRADE recommendation strength) to both HCWs and lay audiences, and the tension between simplifying language and preserving scientific accuracy.
Suggestions
• Shorten and sharpen the Discussion:
o Begin with a clear statement of the 3–4 most important findings (e.g., navigation issues, confusion about recommendation strength and target populations, value of implementation sections, importance of locally contextualized imagery).
o Then discuss how these findings confirm, extend, or challenge previous studies (e.g., BMJ Rapid Recommendations user-testing in Europe).
• Elaborate more explicitly on how these findings might generalize to other guideline projects in LMICs (including maternal and newborn health), not only child health, while acknowledging contextual limits.
• Reflect more deeply on the implications of having some authors both design the infographics and conduct user testing: how might this influence participant feedback or interpretation, and how was this mitigated?

- ***Following your suggestions, we replaced the "Principal findings" section with a new paragraph structured according to four key findings. This also addresses your request to elaborate on how the findings might generalize. The new section reads: "The user testing identified four key considerations for development of CPG summary infographics. First, the importance of clear visual hierarchy and***

prominent placement of recommendations. Second, the importance of using accessible language without losing the intended meaning. Third, to not only communicate what is recommended but to also make the implications for practice readily accessible. Fourth, ensuring that illustrations are context appropriate, as visual elements can influence how users engage with and interpret the content. These findings provide practical insights for developing CPG dissemination products for HCWs in resource-constrained settings."

- ***The section "Comparison with current litterature" already discussed how these findings confirm, extend, or challenge previous studies, but we have shortened it significantly to make it mor concise.***
- ***We agree that the involvement of the lead interviewer in both infographic development and user testing may have influenced the study. Participants may have been more inclined to provide positive feedback if they perceived the interviewer as being closely involved in the project, while the interviewer's familiarity with the design process and intended messages may also have influenced the interpretation of participants' comments. We have therefore revised the "Discussion" to acknowledge both the potential influence on participant responses and the potential for interpretive bias. We also clarify that the interviewer's role in infographic development was not disclosed to participants, which may have reduced the likelihood that participants tailored their feedback specifically to the designer. At the same time, we acknowledge that this did not eliminate the possibility of bias. The interviewer's close involvement also had a practical advantage, as it enabled direct engagement with end users and facilitated the translation of their feedback into subsequent infographic revisions. We now explicitly recognise this trade-off as a limitation of the study. We added further reflections under "Discussion">>"Study limitations", see comment below.***

Strengths and limitations
• The "Strengths and limitations" box is clear and appropriately located; it notes the think-aloud approach, multi-country sample, and topic diversity, as well as the small sample and limited transferability.
• Limitations related to potential social desirability bias, Hawthorne effect, and selection bias (participants recruited via guideline groups) are noted but could be briefly elaborated.
Suggestions
• Clearly state that most participants were healthcare workers involved in tertiary hospitals and academic settings; explain that this might restrict the relevance of findings to district-level facilities or community health workers.
• Include a limitation about using AI-generated images, noting that it could cause minor contextual mismatches and that future research should focus on more participatory image creation. With end users.

- ***As mentioned above, we added a paragraph about the lead interviewers' involvement in design of the infographics: "The involvement of TLH in both the design and user testing of infographics is a potential source of bias. However, her involvement in the infographic design was not communicated to participants. Moreover, her ability to talk to end users directly instead of relying on feedback***

collected by others strengthened our ability to implement the feedback in the final revisions."

- ***Regarding the transferability of findings, we expanded the first paragraph under "Study limitations": "The transferability of our findings is limited by the small number of infographics tested which focused on a narrow range of clinical topics, and by the user-testing being confined to a few urban tertiary-hospitals and academic settings, with no participation from rural areas. HCWs in rural settings may face different challenges and have different information needs from those represented in our sample."***
- ***Your comment about AI generated images is addressed under the "Implications for further research and development" section: "Developing user-friendly infographics adds to the workload of CPG panels, and access to design expertise and co-creation processes may be limited. Although AI tools may help reduce this burden, they can also introduce contextual mismatches. Further research is therefore needed to understand how AI can best support infographic development."***

Conclusions and implications
• The conclusions correctly emphasise that CPG infographics can be useful quick-reference tools, but issues such as navigation, layout, recommendation visibility, and clarity about recommendation strength and target population require attention.
• There is a useful observation that stakeholder engagement should begin earlier in the guideline development process, not only during dissemination and product design.
Suggestions
• Make the conclusions more action-oriented:
o Highlight 3–4 concrete design recommendations (e.g., "place key recommendations and target population information at the top; minimise jargon and clarify intervention/comparator; ensure imagery is co-designed with local HCWs; use smartphone-friendly formats and clear links to full guidelines").
• Consider adding a short paragraph on next steps: whether there are plans to evaluate real-world use of the refined infographics in clinical practice, or to extend the approach to other guideline topics (including maternal and newborn care).

- **Following you suggestion above to shorten the discussion, we have replaced the "Implications for further research and development" section with a condensed version.**
- **Similarly, we have replaced the discussion with a more concise text that contains concrete recommendations.**

Specific BMJ Open style recommendations
1. Patient and public involvement (PPI)
o You appropriately note that patients/public were not involved and justify this.
o Consider adding a brief reflection on whether future work might benefit from including parents/caregivers in infographic design for patient-facing versions, even if beyond the current scope.

We have moved and modified the following sentence from the "Study limitations" section to the "Implications for further research and development section:

“Although we developed infographics for different target audiences, we lacked the capacity to conduct user-testing with policymakers or citizens/guardians/mothers.” We also added: “Future research could explore how the needs and preferences of these groups differ from those of HCWs and how dissemination products could be adapted accordingly.”

2. Data sharing statement
o The current statement “datasets are available from the corresponding author on reasonable request” is acceptable, but BMJ Open often encourages more detail (e.g., what type of data transcripts, coded data, analytic memos can be shared, and with what restrictions).
3. Funding and competing interests
o The funding statement is clear and meets BMJ Open requirements; the role of EDCTP2 is appropriately described.
o Competing interests are reported, but given that several authors co-designed the infographics and led the user testing, consider explicitly framing this as a “non-financial competing interest” and explaining how you attempted to mitigate bias.

- ***We prefer to keep the standard data sharing statement as it is.***
- ***We have updated the competing interests statement to make it more clear: “TLH participated in both the design and user testing of the infographics, which we consider a non-financial competing interest and have acknowledged as a potential bias and study limitation.”***

4. Strength of recommendations vs strength of evidence
o Since confusion around “weak” vs “strong” recommendations is central to your findings, consider inserting a short explanatory note in the Discussion to help readers unfamiliar with GRADE understand the implications.

- ***We have inserted this sentence under “ “Results”>> ”Understandability”: “We retained the recommendation strength labels in the final infographics and explicitly explained them in terms of the balance of benefits and harms and whether most informed people would choose the recommended option.”***
