# Supplementary material for: Optimising summary infographics for clinical practice guidelines on child health: qualitative user testing study with healthcare workers in Malawi, Nigeria and South Africa
Source: BMJ Open. 2026 Oct 5;16(10):e122446. doi: 10.1136/bmjopen-2026-122446 (PMC13646941; doi:10.1136/bmjopen-2026-122446)
Supplement: Supplementary file 4 [file bmjopen-16-10-s002.docx]

## Malawi - Information sheet

**Enhancing evidence-informed guideline recommendations for newborn and young child health in three countries in sub-Saharan Africa: A protocol to assess the impact of the Global Evidence Local Adaptation (GELA) Project activities on effective use of evidence in guideline adaptation/development in Malawi**

| **Name of Principal Investigator** | Dr Nyanyiwe Mbeye |
| --- | --- |
| **Name of Organisation** | Kamuzu University of Health Sciences |
| **Name of Sponsor** | EDCTP |
| **Name of Project and Version** | GELA Project, Version 3.0 |

**Participant information sheet: End user interviews**

We are conducting a study to optimise the dissemination of clinical practice guidelines to healthcare providers in resource constrained settings. You are invited to voluntarily participate in this study because you have been identified as a target end user for clinical practice guidelines.

**Why are we doing this research study?**

Clinical practice guidelines can support healthcare providers to provide the best possible healthcare based on the latest research evidence and other relevant considerations. However, such clinical practice guidelines can only have impact on care if they are effectively disseminated to relevant users. Specifically, healthcare providers must be able to find, access, and understand the recommendations and supporting information in clinical practice guidelines in order to use this in their practice.

In this study, we invite target users (you) to give feedback on clinical practice guideline dissemination products. By listening to your suggestions and feedback, we aim to develop dissemination products that are

understandable and useful for other potential users like you. Ultimately, we hope that this can contribute to better healthcare for more people in your country.

**Who can participate in this research study?**
You must be a health care provider (e.g., medical doctor, midwifes, nurse) in Malawi, Nigeria, and South Africa.

**What will I be asked to do and how much time will it take?**

If you volunteer to participate in this study, we will ask you to take part in an interview that will take approximately 45-60 min. In the interview session, we will present you with examples of clinical practice guideline dissemination products on a topic familiar to you. We will ask about your opinions and preferences, so that we can adjust the dissemination products to better respond to your challenges and needs. We will ask you for permission to video record the interview.

**Will being in this research study help me in any way?**

There are no direct benefits to you for participating in this study. However, there may be some longer-term, indirect benefits to you if the study is able to contribute to enhancing the accessibility, usability, and impact of clinical practice guidelines in your country.

**What are my risks of being in this research study?**

We do not anticipate any risks from participating in this study. If at any time you do not want to answer a particular question, please tell the facilitator and you will not be asked to answer. You are free to not answer any question or speak about any subject that you do not want to. If you feel upset during or after the interview, please tell the interviewer.

**How will my personal information be protected?**

The researchers will not share your name with anyone, and when we write about the research, we will not use your name. All video recordings will be transcribed verbatim, and any identifying information will be redacted from all transcripts. Video recordings will be deleted after the study is completed. Other information from this study, including anonymized transcripts and notes, will be stored electronically using password-controlled software only accessible to the research team. Extracts from your interview may be published in research reports but any direct information that could identify who you are will be removed.

**What happens if I say yes, but I change my mind later?**
Your participation in this research is completely voluntary. You may choose not to take part in the study. If you decide to participate in study, you may stop participating at any time. If you decide not to participate in this study or if you stop participating at any time, you will not be penalised or lose any benefits to which you otherwise qualify.

**Who can I talk to if I have questions?**

Should you have any questions regarding this study and your rights as a research participant or if you wish to report any problems you have experienced related to the study, please contact the Chairperson of the South African Medical Research Council Human Research Ethics Committee (Adri Labuschagne, tel. ************; e-mail: [***********************](mailto:adri.labuschagne@mrc.ac.za)).

## Malawi - Consent form

**Monitoring and Evaluation (M&E) of the Global evidence, local adaptation (GELA) project in Malawi**

| **Name of Principal Investigator** | Dr Nyanyiwe Mbeye |
| --- | --- |
| **Name of Organisation** | Kamuzu University of Health Sciences |
| **Name of Sponsor** | EDCTP |
| **Name of Project and Version** | GELA Project, Version 1.0 |

**Informed consent form: End user interviews**

We are inviting you to participate in an end user test to evaluate whether the ways we have formatted evidence are useful and easy to understand, and how these formats could be improved. This forms part of the larger study which is monitoring and evaluating the Global Evidence - Local Adaptations (GELA) project.

This form is for you to state if you agree to take part in the study. Please read and answer every question. If there is anything you do not understand, or if you would like more information, please ask the researcher.

k

Have you read and understood the information sheet about the study?

Yes ☐	No ☐

Have you had the opportunity to ask questions about the study and have these been answered to your satisfaction?

Yes ☐	No ☐

Do you understand that the study is not designed to help you personally, but that the results may help to improve the relevance and usability of qualitative evidence in evidence-informed decision-making and guideline development and adaptation processes?

Yes ☐	No ☐

Do you understand that your participation is completely voluntary and that you are free not to participate, not to answer a particular question, and to withdraw from the study for any reason, at any time, without any consequences for you?

Yes ☐	No ☐

Do you understand that the information you provide will be held in confidence by the research team?

Yes ☐	No ☐

Do you agree to the use of anonymised/pseudonymised quotes for scientific presentation of the results (for example, academic presentations, reports, or scientific publications)?

Yes ☐	No ☐

Do you agree to take part in the study?

Yes ☐	No ☐

Do you agree to the interview being video recorded, on the condition that any identifying information will not be included in any scientific outputs/publications?

Yes ☐	No ☐

Do agree to other members of the research team being able to view the video recording.

□ yes	□ no

| Name of participant |  | Date |  | Signature |
| --- | --- | --- | --- | --- |
| Name of interviewer |  | Date |  | Signature |

## South Africa - Information sheet

**Monitoring and Evaluation (M&E)
of the Global evidence, local adaptation (GELA) project**

**Participant information sheet: End user interviews**

We are conducting a study to optimise the dissemination of clinical practice guidelines to healthcare providers in resource constrained settings. You are invited to voluntarily participate in this study because you have been identified as a target end user for clinical practice guidelines.

**Why are we doing this research study?**

Clinical practice guidelines can support healthcare providers to provide the best possible healthcare based on the latest research evidence and other relevant considerations. However, such clinical practice guidelines can only have impact on care if they are effectively disseminated to relevant users. Specifically, healthcare providers must be able to find, access, and understand the recommendations and supporting information in clinical practice guidelines in order to use this in their practice.

In this study, we invite target users (you) to give feedback on clinical practice guideline dissemination products. By listening to your suggestions and feedback, we aim to develop dissemination products that are  understandable and useful for other potential users like you. Ultimately, we hope that this can contribute to better healthcare for more people in your country.

**Who can participate in this research study?** 
You must be a health care provider or involved in community child care (e.g., paediatric doctor, midwife, nurse) in Malawi, Nigeria, or South Africa.

**What will I be asked to do and how much time will it take?**

If you volunteer to participate in this study, we will ask you to take part in an interview that will take approximately 45-60 min.  In the interview session, we will present you with examples of clinical practice guideline dissemination products on a topic familiar to you. We will ask about your opinions and preferences, so that we can adjust the dissemination products to better respond to your challenges and needs. We will ask you for permission to video record the interview.

**Will being in this research study help me in any way?**

There are no direct benefits to you for participating in this study. However, there may be some longer-term, indirect benefits to you if the study is able to contribute to enhancing the accessibility, usability, and impact of clinical practice guidelines in your country.

**What are my risks of being in this research study?**

We do not anticipate any risks from participating in this study. If at any time you do not want to answer a particular question, please tell the facilitator and you will not be asked to answer. You are free to not answer any question or speak about any subject that you do not want to. If you feel upset during or after the interview, please tell the interviewer.

**How will my personal information be protected?**

The researchers will not share your name with anyone, and when we write about the research, we will not use your name. All video recordings will be transcribed verbatim, and any identifying information will be redacted from all transcripts. Video recordings will be deleted after the study is completed. Other information from this study, including anonymized transcripts and notes, will be stored electronically using password-controlled software only accessible to the research team. Extracts from your interview may be published in research reports but any direct information that could identify who you are will be removed.

**What happens if I say yes, but I change my mind later?** 
Your participation in this research is completely voluntary.  You may choose not to take part in the study. If you decide to participate in study, you may stop participating at any time. If you decide not to participate in this study or if you stop participating at any time, you will not be penalised or lose any benefits to which you otherwise qualify.

**Who can I talk to if I have questions?**

Should you have any questions regarding this study and your rights as a research participant or if you wish to report any problems you have experienced related to the study, please contact the Chairperson of the South African Medical Research Council Human Research Ethics Committee (Adri Labuschagne, tel. ************; e-mail: [***********************](mailto:adri.labuschagne@mrc.ac.za)).

## South Africa – Consent form

**Monitoring and Evaluation (M&E)**

**of the Global evidence, local adaptation (GELA) project**

**Informed consent form for End user interviews**

We are inviting you to participate in an end user test to evaluate whether the ways we have formatted evidence are useful and easy to understand, and how these formats could be improved. This forms part of the larger study which is monitoring and evaluating the Global Evidence - Local Adaptations ([GELA](https://africa.cochrane.org/projects/global-evidence-local-adaptation-gela)) project.

This form is for you to state if you agree to take part in the study. Please read and answer every question. If there is anything you do not understand, or if you would like more information, please ask the researcher.

Have you read and understood the information sheet about the study?

Yes ☐	No ☐

Have you had the opportunity to ask questions about the study and have these been answered to your satisfaction?

Yes ☐	No ☐

Do you understand that the study is not designed to help you personally, but that the results may help to improve the relevance and usability of qualitative evidence in evidence-informed decision-making and guideline development and adaptation processes?

Yes ☐	No ☐

Do you understand that your participation is completely voluntary and that you are free not to participate, not to answer a particular question, and to withdraw from the study for any reason, at any time, without any consequences for you?

Yes ☐	No ☐

Do you understand that the information you provide will be held in confidence by the research team?

Yes ☐	No ☐

Do you agree to the use of anonymised/pseudonymised quotes for scientific presentation of the results (for example, academic presentations, reports, or scientific publications)?

Yes ☐	No ☐

Do you agree to take part in the study?

Yes ☐	No ☐

Do you agree to the interview being video recorded, on the condition that any identifying information will not be included in any scientific outputs/publications?

Yes ☐	No ☐

Do agree to other members of the research team being able to view the video recording.

□ yes    □ no

In accordance with the provisions of the **Protection of Personal Information Act 4 of 2013** (as amended), I hereby consent:

a.	To my personal information (hereinafter 'data') being collected, processed, shared and stored in accordance with the research protocol as approved by the South African Medical Research Council's Human Research Ethics Committee (SAMRC HREC);

b.	To my anonymised data being shared, processed and transferred by third parties and between third parties, and where relevant beyond the jurisdictional borders of South Africa;

c.	To all findings and results flowing from my anonymised data being broadly shared and published on the conclusion of the research.

| Name of participant (please print) |  | Date |  | Signature |
| --- | --- | --- | --- | --- |
| Name of interviewer (please print) |  | Date |  | Signature |

## Nigeria - Information sheet

**Participant information sheet**

We are conducting a study to optimise the dissemination of clinical practice guidelines to healthcare providers in resource constrained settings. You are invited to voluntarily participate in this study because you have been identified as a target end user for clinical practice guidelines.

**Why are we doing this research study?**

Clinical practice guidelines can support healthcare providers to provide the best possible healthcare based on the latest research evidence and other relevant considerations. However, such clinical practice guidelines can only have impact on care if they are effectively disseminated to relevant users. Specifically, healthcare providers must be able to find, access, and understand the recommendations and supporting information in clinical practice guidelines in order to use this in their practice.

In this study, we invite target users (you) to give feedback on clinical practice guideline dissemination products. By listening to your suggestions and feedback, we aim to develop dissemination products that are understandable and useful for other potential users like you. Ultimately, we hope that this can contribute to better healthcare for more people in your country.

**Who can participate in this research study?**
You must be a health care provider (e.g., medical doctor, midwifes, nurse) in either Malawi, Nigeria, or South Africa.

**What will I be asked to do and how much time will it take?**

If you volunteer to participate in this study, we will ask you to take part in an interview that will take approximately 45-60 min. In the interview session, we will present you with examples of clinical practice guideline dissemination products on a topic familiar to you. We will ask about your opinions and preferences, so that we can adjust the dissemination products to better respond to your challenges and needs. We will ask you for permission to video record the interview.

**Will being in this research study help me in any way?**

There are no direct benefits to you for participating in this study. However, there may be some longer-term, indirect benefits to you if the study is able to contribute to enhancing the accessibility, usability, and impact of clinical practice guidelines in your country.

**What are my risks of being in this research study?**

We do not anticipate any risks from participating in this study. If at any time you do not want to answer a particular question, please tell the facilitator and you will not be asked to answer. You are free to not answer any question or speak about any subject that you do not want to. If you feel upset during or after the interview, please tell the interviewer.

**How will my personal information be protected?**

The researchers will not share your name with anyone, and when we write about the research, we will not use your name. All video recordings will be transcribed verbatim, and any identifying information will be redacted from all transcripts. Video recordings will be deleted after the study is completed. Other information from this study, including anonymized transcripts and notes, will be stored electronically using password-controlled software only accessible to the research team. Extracts from your interview may be published in research reports but any direct information that could identify who you are will be removed.

**What happens if I say yes, but I change my mind later?**
Your participation in this research is completely voluntary. You may choose not to take part in the study. If you decide to participate in study, you may stop participating at any time. If you decide not to participate in this study or if you stop participating at any time, you will not be penalised or lose any benefits to which you otherwise qualify.

**Who can I talk to if I have questions?**

Should you have any questions regarding this study and your rights as a research participant or if you wish to report any problems you have experienced related to the study, please contact the Principal Investigator, Dr. Emmanuel Effa (****************, [***************************](mailto:emmanueleffa@yahoo.com)) or the Chairman of National Health Research Ethics Committee of Nigeria Committee at chairman@nhrec.net, OR secretary@nhrec.net, OR deskofficer@nhrec.net OR +234-523-8367

## Nigeria – Consent form

**Informed consent form**

We are inviting you to participate in an end user test to evaluate whether the ways we have formatted the guideline summaries are useful and easy to understand, and how these formats could be improved. This forms part of the larger study which is monitoring and evaluating the Global Evidence - Local Adaptations (GELA) project. This form is for you to state if you agree to take part in the study. Please read and answer every question. If there is anything you do not understand, or if you would like more information, please ask the researcher.

Have you read and understood the information sheet about the study?

Yes ☐	No ☐

Have you had the opportunity to ask questions about the study and have these been answered to your satisfaction?

Yes ☐	No ☐

Do you understand that the study is not designed to help you personally, but that the results may help to improve the relevance and usability of qualitative evidence in evidence-informed decision-making and guideline development and adaptation processes?

Yes ☐	No ☐

Do you understand that your participation is completely voluntary and that you are free not to participate, not to answer a particular question, and to withdraw from the study for any reason, at any time, without any consequences for you?

Yes ☐	No ☐

Do you understand that the information you provide will be held in confidence by the research team?

Yes ☐	No ☐

Do you agree to the use of anonymised/pseudonymised quotes for scientific presentation of the results (for example, academic presentations, reports, or scientific publications)?

Yes ☐	No ☐

Do you agree to take part in the study?

Yes ☐	No ☐

Do you agree to the interview being video recorded, on the condition that any identifying information will not be included in any scientific outputs/publications?

Yes ☐	No ☐

Do agree to other members of the research team being able to view the video recording.

| Name of participant (please print) |  | Date |  | Signature |
| --- | --- | --- | --- | --- |
| Name of interviewer (please print) |  | Date |  | Signature |

Yes ☐	No ☐
