# Supplementary material for: Optimising summary infographics for clinical practice guidelines on child health: qualitative user testing study with healthcare workers in Malawi, Nigeria and South Africa
Source: BMJ Open. 2026 Oct 5;16(10):e122446. doi: 10.1136/bmjopen-2026-122446 (PMC13646941; doi:10.1136/bmjopen-2026-122446)
Supplement: Supplementary file 5 [file bmjopen-16-10-s003.docx]

Interview guide for GELA infographic user testing

**Interview details**

| Participant number |  |
| --- | --- |
| Location |  |
| Infographic topic and version |  |
| Participant age and sex |  |
| Date of interview |  |
| Interviewer/Observer |  |

**Checklist**

| Interviewer/facilitator | |
| --- | --- |
| Consent forms (printed) |  |
| Interview guide (printed) |  |
| A tablet or laptop with access to the dissemination product |  |
| Paper prints of all layers in the dissemination product |  |
| Gråpgræipg Clock/stop watch too keep time |  |
| Observer | |
| Copy of the interview guide |  |
| Laptop for notetaking (and backup note taking sheet and pen) |  |
| Audio recorder (phone + voice recorder), camera, extra batteries/power bank |  |
| Test equipment before start |  |
| Put “do not disturb, recording in progress” note on the door (bring paper & tape, or large post-it notes) |  |
| Local organizer | |
| Quiet room with 4 chairs |  |
| Water & beverages |  |

**Introduction**

**Orientation of the guest:**

- Thank you very much for the time you took to sit down with us to discuss dissemination of clinical practise guideline to health care professionals in the context of new born and child health. The disseminated product is in a form of a infographic (guideline summary), are you familiar with this.

We would like to record this meeting with you so that we can go back later and make sure we don’t miss anything. After transcribing the interviews and analysing the results we will delete the recordings.

Your input will be used to help us make changes to the infographic before it is officially published next year.

- **Do you have any questions?**
- **In connection with this we have these consent form and can give a few minutes to read these and fill out**

**Start video/audio recording**

As part of the interview we will start with brief introductions of ourselves (I start of by introducing myself, I am Tandekile Lubelwan Hafver I work as a senior researcher at MAGIC Evidence Echosystem Foundation, where we have partnered up with the GELA project for adaptation and updating of child health guidelines

**GELA description**
GELA stands for global evidence local adaptation, where the project aiming’s to enhance the health of newborns and children in sub-Saharan Africa, specifically in Malawi, Nigeria, and South Africa.
The activities in GELA started by reviewing current clinical guidelines in the regions, to find any gaps in clinical guidance. We then collaborated with health ministries and local stakeholders to determine three priority topics in each country, where the clinical practise guidelines for the identified topics needs to be updated.
Local expert teams then examined the most recent research evidence and guidelines on narrowed down topics worldwide. A big strategy within GELA is to minimize research waste and duplication of effort as research group are often found working in silos (in isolation only to find years later they could have collaborated as they are working on the same question. For adaptation it meats the challenges highlighted above by not necessary have denovo guidelines synthesis (make guidelines from scratch). This are very recourse intense. By adapting guidelines recommendations to fit the local cultural and socio-economic conditions.

For our guidelines to have impact, we need to ensure they reach the right people. Typically, clinical practice guidelines are shared as journal papers, but not everyone has access to the articles or read them. So, we're exploring alternative ways to share clinical practise guidelines, including the use of infographics (guideline summaries) for a more visual and concise presentation, and using posters and social media for wider distribution.

- **Goal**
  The purpose of this session is for us to learn how we can make the infographic more user-friendly and useful for potential users like you. followed by a brief description of the GELA project.
- The goal of the session is for us to learn how we can make the infographic more user-friendly and useful for potential users like you.

What you can expect in the interview: I will serve as the interviewer, Valerie ans Susan will serve as observers and will also decode the insight from the meeting. **Your role is to let us know what you are thinking.** We will start by asking you some general questions about you and how you use research and guidelines in your work. Next, we will ask you to look at an infographic that we have developed for 10 seconds or so. To determine your first thoughts and first impressions of the product, through asking you a few questions. After this we will take a deeper look at the infographic and ask more specific questions that we would like feed-back on.
Throughout the session, we encourage you to “think out loud” about what you are doing and looking at.

**Remember that we are testing the product and not you, so no answer is wrong.** If you run into any problems, it’s not your fault and it means there’s something we need to change – in the content, the design, or maybe our whole approach. If you run into trouble, don’t worry - this is actually helpful for us.

You are here because you know what it’s like to be a healthcare provider looking for health care information, so your perspective will help us make this product more useful. I (Tandi) will be facilitating this session.
Valerie will be observing and taking notes. 
XX is also here as our local observer.

**Your role is just to let us know what you are thinking.** 
We will start by asking you som general questions about you and how you use research and guidelines in your work.
Next, we will ask you to look at an infographic that we have developed. We will let you explore it and ask you about your immediate first impressions before we give you more specific questions that we would like feed-back on.
Throughout the session, we encourage you to “think out loud” about what you are doing and looking at.

**Remember that we are testing the product and not you, so no answer is wrong.** If you run into any problems, it’s not your fault and it means there’s something we need to change – in the content, the design, or maybe our whole approach. If you run into trouble, don’t worry - this is actually helpful for us.

You are here because you know what it’s like to be a healthcare provider looking for health care information, so your perspective will help us make this product more useful.

**We really appreciate having you here to help us out.**

| **Greeting and introduction** | Welcome users, offer beverage  Introduce yourself and others  Explain the background of the project  Explain the goals of the session  Give them informed consent form, get signature | **GELA description:**  We're part of a project named GELA, aiming to enhance the health of newborns and children in sub-Saharan Africa, specifically in Malawi, Nigeria, and South Africa. In this project, we started by reviewing current clinical guidelines in the region, to find any gaps. We then collaborated with health ministries and local stakeholders to determine three priority topics in each country that need updated clinical practice guidelines. Local expert teams then examined the most recent research and guidelines on these topics worldwide. They adapted these recommendations to fit the local cultural and socio-economic conditions. For our guidelines to have impact, we need to ensure they reach the right people. Typically, clinical practice guidelines are shared as journal papers, but not everyona have access to them or read them. So, we're exploring alternative ways to share them, including infographics for a more visual and concise presentation, and using posters and social media for wider distribution.  **Goal** The purpose of this session is for us to learn how we can make the infographic more user-friendly and useful for potential users like you. |
| --- | --- | --- |
| **Explain roles** | Explain the different roles  Explain what’s expected of them  Remind them of their qualifications  Explain that you’re testing the interface, not them.  Reassure users about what will happen if they encounter any difficulties  Reiterate how valuable this is and how much you appreciate their help | I (Tandi) will be facilitating this session.  Valerie will be observing and taking notes.  XX is also here as our local observer.  **Your role is just to let us know what you are thinking.**  We will start by asking you som general questions about you and how you use research and guidelines in your work.  Next, we will ask you to look at an infographic that we have developed. We will let you explore it and ask you about your immediate first impressions before we give you more specific questions that we would like feed-back on. Throughout the session, we encourage you to “think out loud” about what you are doing and looking at.  **Remember that we are testing the product and not you, so no answer is wrong.** If you run into any problems, it’s not your fault and it means there’s something we need to change – in the content, the design, or maybe our whole approach. If you run into trouble, don’t worry - this is actually helpful for us.  You are here because you know what it’s like to be a healthcare provider looking for health care information, so your perspective will help us make this product more useful.  **We really appreciate having you here to help us out.** |
| **They are in charge** | Confirm ending time and that they can stop or take a break  Ask permission to record and explain what will happen with the data | We would like to record this meeting with you so that we can go back later and make sure we don’t miss anything. After transcribing the interviews and analysing the results we will delete the recordings.  Your input will be used to help us make changes to the infographic before it is officially published next year.  **Do you have any questions?** |
| **Start video/audio recording** | | |

**Ice breaker questions:**

1) Could you describe your current role and responsibilities in your position?

| How do you see your role? | |
| --- | --- |
| Paediatrician  Nurse  Dietitian  Other | □ yes    □ no  □ yes    □ no  □ yes    □ no  □ yes    □ no  Describe: |

2) What has been your journey and years of experience in clinical practice? (Do you have academic experience beyond medical school? (e.g. PhD, PostDoc, other))

| What is your educational background and your current position? | |
| --- | --- |
| Medical background  Research background  Administration background  Other | □ yes    □ no  □ yes    □ no  □ yes    □ no  □ yes    □ no  Describe: |
| Current position: | |

3) What do you usually do to find answers when you're not sure about something in your work? (e.g. ask colleagues, look in textbooks, check clinical guidelines, Google, other?)

4) Do you use clinical practice guidelines? Why/why not? How often? In what situations?

5) What are your preferred devices and channels for accessing clinical information? (e.g. journals, books, phone, tablet, computer) How do these choices fit into your daily routine?

Other notes/first impressions from this section

**First impression (show on screen)**

Show the page for healthcare providers for 10 seconds then hide.

6) What do you think this is about? Who is it for? (Are you familiar with this topic?)

7) What was the first thing that caught your eye?

8) Any other features you noticed?

Other notes/first impressions from this section

**Open exploration of product**

Go to the landing page.

9) First, we would like you to look around and explore the infographic like you would without us in the room. Remember to think out loud, explain what you are looking at, what you expect to find, and what you think.

**Guided exploration**

Go to landing page

10) What is this section showing? Please comment about what you see.

11) (Go to page for healthcare providers) What is this section showing? Please comment about what you see, e.g. what you would expect to see and if anything confusing or missing.

12) (Go to “Background information”) What is this section showing? Please comment about what you see, e.g. what you would expect to see and if anything confusing or missing.

13) (Go to “The patients this recommendation applies to”) What is this section showing? Please comment about what you see, e.g. what you would expect to see and if anything confusing or missing.

14) (Go to “Recommendation”) What is this section showing? Please comment about what you see, e.g. what you would expect to see and if anything confusing or missing.

15) (Go to “Considerations for hospital staff”) What is this section showing? Please comment about what you see, e.g. what you would expect to see and if anything confusing or missing.

16) (Go to “How this recommendation was made”) What is this section showing? Please comment about what you see, e.g. what you would expect to see and if anything confusing or missing.

Other notes/first impressions from this section

**Specific questions based on Honeycomb model**

17) (**Useful**): Would this kind of infographic be useful for you if you were going to make a decision about care? (How/why?)

18) (**Usable**) Could you comment on the user-friendliness? (How intuitive Is the layout and format? How would it work on your preferred device?)

19) (**Understandable**) In terms of clarity and comprehension, can you explain how easy or difficult it is to understand the information in the infographic? (Does it give you the answers you need?)

20) (**Accessible**) How do you think people would like to access this? (e.g. webpage, email, WhatsApp) How could we reach more people?

21) (**Credible**) Would you trust this information? (Why/why not?)

22) (**Desirable**) To the degree you can ”like” a presentation of information, did you like this table or not like it? (Possible probes: What do you think of the content, language formatting?)

23) (**Valuable**) Overall, do you think this type of presentation would be valuable for you or other people like you? (If not clear already, can you explain why?)

Other notes/first impressions from this section

**Suggestions for improving the product**

24) If there are any specific types of information you believe should be included or emphasised further in this product, can you explain them

25) Can you comment on any elements in this product that you think are superfluous (surplus; redundant, unnecessary,unessesntial, unneeded or confusing?

26) Regarding the visual appeal, what colour scheme or design elemnts do you think could enhance the product's utility and attractiveness for its users?

27) If given the opportunity, what modifications or enhancements would you make to this product? Why?

Other notes/first impressions from this section

**Suggestions for improving the user testing**

28) Do you have any feedback to us on how we have conducted this session with you? (for instance the information you received, or how we carried out the interview)

Thank you very much, that was all, we are finished.

**Stop video/audio recording**

**After interview:**

Note down important points raised and first impressions from the interview
